# Identification of crude drugs in the Japanese pharmacopoeia using a DNA barcoding system

**DOI:** 10.1038/srep42325

**Published:** 2017-02-10

**Authors:** Xiaochen Chen, Li Xiang, Linchun Shi, Gang Li, Hui Yao, Jianping Han, Yulin Lin, Jingyuan Song, Shilin Chen

**Affiliations:** 1 Institute of Medicinal Plant Development, Chinese Academy of Medical Sciences & Peking Union Medical College, Key Laboratory of Bioactive Substances and Resources Utilization of Chinese Herbal Medicine, Ministry of Education, Beijing 100193, P.R. China; 2 Institute of Chinese Materia Medica, China Academy of Chinese Medical Sciences, Beijing 100700, P.R. China; 3 China Medico Corporation, Talent International Building, No. 80, Guangqumen Nei Street, Dongcheng District, Beijing 100062, P.R. China

## Abstract

Kampo is the general designation for traditional Japanese herbal medicines, which are recognized as official medicines and listed in the Japanese pharmacopoeia (JP). In most cases, it is difficult to identify the crude drug materials to species level using only traditional identification methods. We report the first online DNA barcode identification system, which includes standard barcode sequences from approximately 95% of the species recorded in the JP (16^th^ edition). This tool provides users with basic information on each crude drug recorded in the JP, DNA barcoding identification of herbal material, and the standard operating procedure (SOP) from sampling to data analysis. ITS2 sequences (*psbA-trnH* was an alternative when ITS2 could not be amplified) were generated from a total of 576 samples to establish the database. An additional 100 samples (from different medicinal parts, from both single origin and multiple origins and from both retailers and the planting base) were identified using the system. A total of 78% of the test samples were identified as the species listed on their label. This system establishes a model platform for other pharmacopeias from countries like China, Korea, the US and the European Union, for the safe and effective utilization of traditional herbal medicines.

Kampo originated from traditional Chinese medicines via Korea and has developed into its own system through thousands of years of clinical practice^1^. Traditional Chinese medicines were first introduced into Japan in the 6^th^ century and were modified according to local natural and cultural circumstances over the next thousand years (until approximately the 17^th^ and 18^th^ centuries, the Edo era). Kampo medicine endured a crisis during the Meiji restoration of the late 19^th^ century, when Western medicine was highly advocated^2^. However, it survived because of its irreplaceable advantages, including low price, convenient sources and clearly effective treatments. Although Western medicine is now the mainstream approach in Japan, many Japanese people still utilize Kampo medicines. They have been used in cancer treatment to help strengthen patients’ physical reconditioning and reduce the side effects of radiotherapy and chemotherapy^3^. The application of Kampo combined with Western medicine is well regarded in Japan because of its effectiveness^4^,^5^, demonstrating the ability to cure chronic diseases such as hypertension, diabetes, and allergic diseases^6^. These irreplaceable advantages drive the development of Kampo medicines with the aim of helping people improve their quality of life.

The first edition of the Japanese pharmacopoeia (JP) was issued in 1886 during the Meiji Era and included 77 European herbs. Unfortunately, it was not until 1974 that traditional Japanese herbal medicines were finally recognized as official medicines^7^. Since the 7^th^ edition, the JP has been revised every five years; the newest revision is the 16^th^ edition, published by the Ministry of Health, Labour, and Welfare. More than 276 items (crude drugs, decoctions and preparations) are listed in the newest edition of the JP^8^. The crude drugs used in Kampo are primarily imported from China and other countries in Southeast Asia, though a small portion are harvested or cultivated in Japan. Historical, geographical and taxonomic differences, along with different academic interpretations and genetic variations have resulted in divergence between traditional Chinese and Japanese herbal medicine. The specific differences include the existence of homonyms and synonyms and differences in the sources and parts used to obtain an identical crude drug^9^. Exchange in traditional medicines has expanded, and more crude materials and preparations are imported and exported between China and Japan as a result. To ensure drug safety and clinical efficacy, an accurate and rapid test to identify the sources of crude drugs is necessary.

Compared with morphological, microscopic, and chemical identification methods for herbal medicines, DNA barcoding is a new tool that has been widely used in taxonomy^10^, biodiversity estimation^11^, phylogenetic studies^12^,^13^, and biological identifications^14^,^15^,^16^,^17^. Barcoding is a DNA-based approach for identifying species that relies on species-level discrimination using a short DNA region. This technique was first introduced by Hebert *et al*. in 2003^14^. Several barcoding candidates have been proposed and compared regarding their identification efficacy. Plastid genes (*rbcL* and *matK*) and the intergenic spacer *psbA-trnH* have been recommended as land plant barcodes^18^,^19^. Traditional medicines are often composed of plant organs. Adulterants and related species are frequently confused in applications, and the previous single or incorporated barcode(s) is far from perfect for medicinal plant identification. Chen *et al*. tested the discrimination ability of ITS2 in more than 6600 plant samples and then recommended ITS2 as a novel DNA barcode for identifying medicinal plant species^20^. In 2011, the China Plant BOL Group analyzed the effectiveness and universality of different combinations of DNA barcodes based on a large dataset (6286 individuals representing 1757 species in 141 genera of 75 families). These samples were tested using different methods of data analysis. The results suggest that “ITS/ITS2 should be incorporated into the core barcode for seed plants”^21^. A complete barcoding identification system for herbal material, from sampling to data analysis, has been developed using the ITS2 region together with the *psbA-trnH* region^22^. Kool *et al*.^23^ used DNA barcodes to test commercialized medicinal plants from southern Morocco. The successful identification of crude drugs such as *Panax*^24^, *Rhodiola* products^25^, and caterpillar fungus^26^ has demonstrated that DNA barcoding can be effectively used as a new authentication method for traditional medicine. This technique will promote the safety and efficiency of clinical applications and market circulation.

In this study, we established a standardized barcode identification system for crude Kampo materials, and the results indicated that DNA barcoding is an efficient tool for traditional herbal medicine authentication.

## Results

### PCR amplification and sequencing success rate

First, all samples were amplified using the ITS2 primer pairs. From a total of 576 samples, 517 were successfully amplified and sequenced, corresponding to a success rate of 89.8%. The remaining 59 samples were amplified using the *psbA-trnH* primer pairs. Forty-eight of the 59 samples were generated with *psbA-trnH* sequences, representing an amplification and sequencing success rate of 81.4%. A total of 565 ITS2 and *psbA-trnH* sequences were obtained from the 576 collected samples, a percentage of 98.1%. Five species failed to yield ITS2 or *psbA-trnH* sequences (*Achyranthes fauriei, Jateorhiza columba, Elettaria cardamomum, Aconitum japonicum* and *Digenea simplex*). The failure of DNA isolation and PCR amplification may be attributed to the long-term storage period of some samples and the low degree of matching between the universal primers and the particular species.

### Intra- and inter-species genetic distances of ITS2 and *psbA-trnH*

Intra- and inter-specific (the number of intra-specific samples ≥ 2 and the number of congeneric species ≥ 2) genetic distances of each species were calculated using both K2P and P models. K2P model is one of the models of DNA evolution analysis. It corrects for multiple hits, taking into account transitional and transversional substitution rates, while assuming that the four nucleotide frequencies are the same and that rates of substitution do not vary among sites. P distance is the proportion (p) of nucleotide sites at which two sequences being compared are different. It is obtained by dividing the number of nucleotide differences by the total number of nucleotides compared. It does not make any correction for multiple substitutions at the same site, substitution rate biases (for example, differences in the transitional and transversional rates), or differences in evolutionary rates among sites (quoted from Instruction of Mega software). Average maximum intra-specific distances of ITS2 sequences were 0.0063 and 0.0062 from K2P and P models, respectively. Average minimum inter-specific distances of ITS2 sequences were 0.0221 and 0.0206 from K2P and P models, respectively. Average maximum intra-specific distances of *psbA-trnH* sequences were 0.0034 and 0.0033 from K2P and P models, respectively. Average minimum inter-specific distances of *psbA-trnH* sequences were 0.0026 from both K2P and P models. Besides, nucleotides variations (NV) (InDels were considered when calculating the NV) were also calculated at both intra- and inter-specific levels (Table 1).

### Establishment of a barcode database for crude drugs recorded in the Japanese pharmacopoeia

The herbal material identification system (www.jpbarcode.com) is based on the ITS2 region together with the *psbA-trnH* region and includes standard sequences from approximately 95% of the species recorded in the JP (16^th^ edition) (Table 2). A total of 538 ITS2 sequences (517 from our experiment and 21 from GenBank) and 50 *psbA-trnH* sequences (48 from our experiment and two from GenBank) were included in the JP database. Three sequences from different samples were included for each species. Only 10 species had fewer than three sequences. This database provides users with the following three functions:

1. Basic information on each crude drug recorded in the JP;
2. Herbal material DNA barcoding identification;
3. The standard operating procedure (SOP) from sampling to data analysis.

### Basic information on crude drugs

The first section of the database provides basic information on the crude drugs in the pharmacopoeia. The title of each item includes the English name, the Latin name and the Japanese name of each crude drug. Three components are included for each item: the origin, sequence obtained and description. The origin describes where the crude drug originated (original plant) and the medicinal parts of the plants (the organs utilized as medicines according to historical records, e.g., roots, leaves, fruits) used in clinical applications (referring to the JP). The procedures employed to obtain the sequences are listed next. The detailed process, from material preparation to PCR amplification, is described. For most materials, the SOP was followed (described in the *Standard operating procedure from sampling to data analysis* section). Any improvements or extra steps are recorded in this section. The last part of this section consists of the description of the crude drug quoted from the JP. This section describes the morphological characteristics of the crude drug and its taste and smell, which are important evaluation indexes in the traditional medicine system.

### Herbal material DNA barcoding identification

This section is the core of the database. Both annotations and identification are provided in this section. Users enter sequences in FASTA format and then submit them for analysis. The identification function is based on the BLASTn algorithm. The search results will display one or more species with the highest similarity as the identification result. Detailed information regarding the sequence ID, species name, score, E-value and alignment results are also displayed on the output page.

### The standard operating procedure (SOP) from sampling to data analysis

The database provides users with a DNA barcoding SOP from sampling to data analysis.

#### Sample collection

Techniques for preparing the collected samples and optimal storage conditions are described to ensure accurate identification results.

#### DNA extraction

The general steps for DNA extraction and the processing of different medicinal parts are summarized. For example, polyvinylpyrrolidone (PVP) and mercaptoethanol must be used when grinding the materials because of the high contents of polysaccharides and polyphenols in root- and rhizome-based crude drugs, and acetone can be helpful in DNA isolation from fruit and seed materials to remove liposoluble phenolics.

#### PCR amplification and sequencing

The universal primers and conditions used for PCR are listed in this section. Sequencing in both directions is required for the purified PCR products.

#### Sequence assembly

The generated raw sequences must be estimated based on their quality and according to the specified parameters (listed on the page) before assembly. The ITS2 region is annotated to remove 5.8 S and 28 S sequences based on the HMM. The assembled sequences are then entered into the identification page.

### A case study of the utility of the database

One hundred crude drug and plant materials from 66 different species were collected from a drugstore (52 samples), several botanical gardens in Japan (7 samples), and a Chinese medicine company in China (41 samples) as test samples. ITS2 sequences were obtained following standard procedures. We found that 78 out of the 100 test samples were identified as the labeled species. The remaining 22 test samples were correctly identified to the genus level (see Supplementary file Table S2). These genera included *Angelica, Chrysanthemum, Clematis, Ephedra, Epimedium, Gentiana, Perilla,* and *Prunus*.

## Discussion

### The first identification system including standard ITS2 and *psbA-trnH* experimental sequences and the SOP for DNA barcoding for the identification of crude Kampo drugs

This system constitutes an important contribution to the safe and effective use of traditional herbal medicines in both Japan and China. The relationships and differences between the JP and the Chinese pharmacopoeia (CP) necessitate a complete system for identifying crude herbal drugs. According to our statistical analysis, approximately 66% of the original plants are identical between the JP and the CP. However, because of geographical and cultural variations, there are differences, including the existence of homonyms and synonyms and differences in the sources and medicinal parts utilized for identical crude drugs between the two pharmacopoeias. Angelica (Dang Gui) is recorded in both pharmacopoeias. In the JP, the source is *Angelica acutiloba* Kitagawa, whereas the source is *Angelica sinensis* (Oliv.) Diels in the CP. Chemical composition analysis demonstrates that differences exist in the type and content of the volatile oils of these species, which leads to differences in clinical efficacy^27^. The 22 ITS2 sequences of *A. acutiloba* and *A. sinensis* were 230 bp in length after alignment. There were 30 stable variable sites in the ITS2 sequences between these two species (see Supplementary file Table S3). This result demonstrated that ITS2 is an excellent tool for the identification of different origins of Angelica roots. Three different origins consist of Scopoliae Rhizoma (*Scopolia carniolica, Scopolia japonica*, and *Scopolia parviflora*). Although the species come from the same genus *Scopolia*, they could be clearly discriminated using ITS2 region by constructing Neighbor-joining tree (see Supplementary file Fig. S1). Trade contacts in traditional medicine have been increasingly frequent. A majority of the herbal crude Kampo drugs in Japan are imported from China, and many Kampo companies choose to set up their planting bases in China. A selection of patented Kampo medicines from Japan are also exported to China. To ensure the accurate application of Kampo medicines, beginning at the origin of cultivation and extending to clinical uses, a complete herbal medicine identification system is essential. Genetic information-based DNA barcoding has several advantages over traditional morphological, microscopic, and chemical identification. Genetic information typically remains stable during the entire life of an individual, making DNA a reliable marker for species identification with high repeatability and versatility. Additionally, constructing the SOP for DNA barcoding using a unified database and identification platform will aid in the development of a rapid and accurate herbal material authentication system^25^. Recent reports confirm that DNA barcoding is a powerful tool for the identification of multiple ingredients using next-generation sequencing platform technology^28^,^29^. This technology will enable characterization of the raw materials in traditional patent herbal medicines.

DNA barcoding has initiated a renaissance in herbal medicine identification^22^. Recently, researchers used DNA barcodes to authenticate herbal drugs in the Ayurvedic Pharmacopoeia (AP)^30^. The outstanding study developed a quality control protocol for medicinal plant raw drugs by incorporating DNA barcoding, which had significant meaning of drug safety to the consumers. Here differences were compared between the two studies that both focused on the official Pharmacopoeias. Firstly, there were only 15 species identical to our study according to the Latin names (with a percentage of 7.3%). Secondly, in our study ITS2 and *psbA-trnH* region were used to identify herbal medicines, while *rbcL* region were used in the Ayurvedic Pharmacopoeia study. Thirdly, compared to the Ayurvedic Pharmacopoeia study, in which a reference DNA barcode library was created, we established an online system with multiple functions. Fourthly, both in these two studies, 100 test samples were collected to be identified using its own established database. In the Ayurvedic Pharmacopoeia study, 100 test samples included samples of seeds and powders, while in our study we chose the 100 test samples representing crude drugs: i) originating from different medicinal parts (including the roots, stems, leaves, flowers, fruits, and seeds); ii) from both a single origin and multiple origins; iii) from both retailers and the planting base. In the end, we compared the identification efficiency at species level of the 100 test samples from these two studies. At first, we counted the total involved species of the test samples respectively and then excluded the duplicated samples of each species (in other words, only one sample of the same species was selected to statistic the identification efficiency if the identification results of different samples were the same). Finally, we got a result of 81.8% from our study (54 out of 66) and 76.7% (69 out of 90) from the Ayurvedic Pharmacopoeia study (see Supplementary file Table S4).

Traditional herbal medicines show great potential for more widespread use, in addition to facing challenges. Traditional herbal medicines are welcomed in modern society, particularly among people with suboptimal health, chronic diseases^31^,^32^,^33^,^34^ and obesity^33^ and patients undergoing cancer treatment, in whom herbal medicines are used to strengthen the effects and reduce the side-effects of radio- and chemotherapy^35^. However, poor quality of herbal products and inaccurate herbal medicine applications can lead to several problems (Zhao *et al*., 2008). Newmaster *et al*.^36^ used DNA barcoding to test the quality of herbal products in North America and concluded that most herbal products were of poor quality, revealing the lack of supervision of the herbal products market. Mishra *et al*.^37^ also proposed that DNA barcoding would be important in the development of herbal-based health care as an efficient tool for overcoming authentication challenges. Two medicinal *Aconitum* species (*Aconitum japonicum* and *Aconitum carmichaelii*) are recorded in the JP. Many incidents arising from mistakes regarding *Aconitum* toxicity or the improper clinical application of these species have been reported^38^,^39^. These cases reflect the toxicological problems related to herbal medicine. A DNA barcoding system would be an effective and accurate authentication method for herbal medicines and will aid in ensuring crude drug safety. Our system sets up a model platform for the other pharmacopeias in countries such as China, Korea and the US as well as the European Union, helping to ensure the public medication safety of traditional herbal medicines.

### A better understanding of the authentication results for one hundred test samples using our database

In the case study, 78% of the test samples were authenticated as the labeled species. However, 22% of the test samples were not identified to the species level using our database. Origin information was retrieved using scientific names to better understand the ambiguous identification results. *Perilla frutescens* var. *crispa* and *Perilla frutescens* var. *acuta* are accepted as the subspecies of *Perilla frutescens*. Other cases of ambiguous identification at the species level were the result of close relationships, such as that between *Chrysanthemum indicum* and *Chrysanthemum morifolium*. Hu^40^ indicated that these species could not be distinguished using the ITS2 region because of the low rate of variation within the genus *Chrysanthemum*. Hu also reported that these two species can be well discriminated based on their whole-chloroplast genomes using a MP tree. Chloroplast genomes have been proposed as an effective tool for species identification and phylogenetic studies. Li *et al*.^41^ discussed the possibility of employing whole-plastid-based barcodes and proposed the use of cp genomes as a super barcode, particularly for closely related species. Parks *et al*.^42^ reconstructed the infrageneric phylogeny of *Pinus* from 37 cp genomes and concluded that whole-cp genomes could increase the phylogenetic resolution at lower taxonomic levels. Among our test samples, similar situations occurred for crude drugs such as Clematis root and Japanese Angelica root, which have multiple origins. The common short DNA regions can not be used as an efficient DNA barcode for these closely related species. Chloroplast genomes may have the potential to discriminate between closely related medicinal species. Based on the findings and context discussed above, we made two main conclusions. First, a system using an ITS2 DNA barcode together with *psbA-trnH* is an effective tool for the identification of crude drug materials listed in the JP. Second, the longer length and increased variation and informative sites of chloroplast genomes may be used for identification when a short DNA barcode is not adequate, particularly for lower taxa and closely related species.

## Methods

### Sample collection

Plant specimens were collected from the botanical gardens of Fukuoka University, the University of Toyama and the University of Tokyo in Japan as well as botanical gardens in different regions and the herbariums of universities and colleges in China. Crude medicinal materials and decoctions were collected from a pharmacy in Tokyo and a Kampo company in Japan. The sources of materials from China included pharmacies, hospitals, herbal medicinal markets and the herbariums of universities and colleges. A total of 576 samples (201 species from 129 genera and 66 families) were collected to establish the database (see details in the Supplementary file Table S1). The original plants from Japan were identified based on morphological characters by local botanists. The original plants from botanical gardens and the crude drug samples from medicinal markets in China were identified morphologically by Prof. Lin Yulin from our institute. Voucher specimens were deposited in the herbarium of the Institute of Medicinal Plant Development, Chinese Academy of Medical Sciences (CAMS) and Peking Union Medical College (PUMC), Beijing, China. All of the sequences generated from the collected samples were verified through BLAST analysis, calculation of K2P distances and construction of phylogenetic trees. When the results from the above three steps were identical, the sequences were used in establishment of the database. An additional one hundred samples were collected to test the database, involving 66 species from 57 genera. They were selected following three principles:

- The samples represented crude drugs originating from different medicinal parts of plants, including the roots (*Aconitum carmichaelii*), stem (*Akebia trifoliata*), leaves *(Epimedium grandiflorum* var. *thunbergianum*), flower (*Chrysanthemum indicum*), fruits (*Schisandra chinensis*), and seeds (*Coix lacryma-jobi* var. *mayuen*).
- The samples represented crude drugs from both a single origin (*Panax ginseng*) and multiple origins (*Clematis hexapetala* & *Clematis manshuria*).
- The samples represented crude drugs from both retailers (the Kampo store in Tokyo) and the planting base (the Kampo joint venture in Jinlin province, China).

### DNA extraction, amplification and sequencing

All materials were wiped with 75% ethanol before genomic DNA was isolated. Approximately 25–60 mg of root, rhizome, stem, cortex, fruit and seed material and 10–20 mg of leaf and flower material were prepared for DNA isolation. The materials were rubbed (30 times/second, two minutes) in a Mixer Mill MM400 (Retsch GmbH, Haan, Germany). A Plant Genomic DNA Kit (Tiangen Biotech (Beijing) Co., Ltd., Beijing, China) was used to extract genomic DNA according to the instruction manual. The ITS2 region could not be successfully obtained for some species because of multiple copies in the genome or a low degree of matching between the universal primers and the species. In these species, *psbA-trnH* sequences were amplified. The universal primers and PCR conditions employed in these assays were described previously^20^,^43^. The purified PCR products were sequenced in both directions using an ABI3730 sequencer (Applied Biosystems, USA).

### Data analysis

Trace files were assembled using CodonCode Aligner version 4.2.1, and ITS2 sequences were subjected to a hidden Markov model (HMM)^44^ analysis to remove the conserved 5.8 S and 28 S rRNA genes. K2P distances were calculated using Mega5.1. In addition to the experimental sequences, another 24 sequences from GenBank were also deposited in the database. Both the experimental and GenBank sequences analyzed in this study were screened through BLAST analysis, calculation of K2P distances and construction of phylogenetic trees to eliminate incorrect sequences (including samples contaminated with fungi or other unrelated species).

## Additional Information

**How to cite this article:** Chen, X. *et al*. Identification of crude drugs in the Japanese pharmacopoeia using a DNA barcoding system. *Sci. Rep.* **7**, 42325; doi: 10.1038/srep42325 (2017).

**Publisher's note:** Springer Nature remains neutral with regard to jurisdictional claims in published maps and institutional affiliations.

## Supplementary Material

Supplementary Information

## Figures and Tables

**Table 1:** Analysis of inter-specific divergence between congeneric species and intra-specific variation of ITS2 and psbA-trnH.

Model	K2P distance		P distance		Nucleotides variation	
DNA Barcode	ITS2	*psbA-trnH*	ITS2	*psbA-trnH*	ITS2	*psbA-trnH*
**Sample size**	285	33	285	33	285	33
**Number of species**	95	11	95	11	95	11
**Average maximum intra-specific distance**	0.0063 ± 0.0134	0.0034 ± 0.0058	0.0062 ± 0.0128	0.0033 ± 0.0057	0.0060 ± 0.0125	0.0031 ± 0.0054
**Average intra-specific distance**	0.0043 ± 0.0090	0.0022 ± 0.0038	0.0042 ± 0.0086	0.0022 ± 0.0038	0.0041 ± 0.0084	0.0020 ± 0.0036
**Average minimum inter-specific distance**	0.0221 ± 0.0412	0.0026 ± 0.0038	0.0206 ± 0.0364	0.0026 ± 0.0038	0.0196 ± 0.0354	0.0022 ± 0.0031
**Average inter-specific distance**	0.0393 ± 0.0447	0.0529 ± 0.0447	0.0365 ± 0.0386	0.0361 ± 0.0288	0.0351 ± 0.0377	0.0272 ± 0.0210

Note: 1. Nucleotides variation = number of variation sites/full length after alignment. InDels were included.

2 Sequence statistics of *psbA-trnH* only retained the partial intergenic region when calculating.

**Table 2:** Coverage of the experimental and GenBank sequences included in this study in reference to the JP.

	No. of crude drugs^a^	No. of species	No. of families	No. of genus
Recorded in the JP	146	211	66	129
Collected in this research	142	200	65	126
Percentage^b^ (%)	97.3	94.8	98.5	97.7

^a^Secretions, extracts, exudates and some processed products are not included.

^b^Percentage = No. in this study/No. in pharmacopeia.

## References

[b1] Matsumoto C. . A proteomic approach for the diagnosis of ‘Oketsu’ (blood stasis), a pathophysiologic concept of Japanese traditional (Kampo) medicine. Evid. Based Complement. Alternat. Med. 5, 463–474 (2008). 18955217 10.1093/ecam/nem049 PMC2586309

[b2] Motoo Y., Arai I., Hyodo I. & Tsutani K. Current status of Kampo (Japanese herbal) medicines in Japanese clinical practice guidelines. Complement. Ther. Med. 17, 147–154 (2009). 19398068 10.1016/j.ctim.2008.09.003

[b3] Efferth T., Miyachi H. & Bartsch H. Pharmacogenomics of a traditional Japanese herbal medicine (Kampo) for cancer therapy. Cancer Genomics Proteomics 4, 81–91 (2007). 17804870

[b4] Ikegami F., Fujii Y. & Satoh T. Toxicological considerations of Kampo medicines in clinical use. Toxicology 198, 221–228 (2004). 15138045 10.1016/j.tox.2004.01.029

[b5] Ikegami F., Sumino M., Fujii Y., Akiba T. & Satoh T. Pharmacology and toxicology of Bupleurum root-containing Kampo medicines in clinical use. Hum. Exp. Toxicol. 25, 481–494 (2006). 16937920 10.1191/0960327106het654oa

[b6] Makino T., Mizuno F. & Mizukami H. Does a kampo medicine containing schisandra fruit affect pharmacokinetics of nifedipine like grapefruit juice? Biol. Pharm. Bull. 29, 2065–2069 (2006). 17015952 10.1248/bpb.29.2065

[b7] Saito H. Regulation of herbal medicines in Japan. Pharmacol. Res. 41, 515–519 (2000). 10753549 10.1006/phrs.1999.0645

[b8] Li Z. & Anwei D. On the subsequent revision of Pharmacopoeia of the People’s Republic of China: volume 1 (2010 edition) - based on the regulation of crude drugs standard in the Japanese Pharmacopoeia (16th edition). J. Nanjing Univ. Trad. Chin. Med. 28, 301–308 (2012).

[b9] Yu F. . Traditional Chinese medicine and Kampo: a review from the distant past for the future. J. Int. Med. Res. 34, 231–239 (2006). 16866016 10.1177/147323000603400301

[b10] Miller S. E. DNA barcoding and the renaissance of taxonomy. Proc. Natl. Acad. Sci. USA 104, 4775–4776 (2007). 17363473 10.1073/pnas.0700466104 PMC1829212

[b11] Lahaye R. . DNA barcoding the floras of biodiversity hotspots. Proc. Natl. Acad. Sci. USA 105, 2923–2928 (2008). 18258745 10.1073/pnas.0709936105 PMC2268561

[b12] Hajibabaei M., Singer G. A., Hebert P. D. & Hickey D. A. DNA barcoding: how it complements taxonomy, molecular phylogenetics and population genetics. Trends Genet. 23, 167–172 (2007). 17316886 10.1016/j.tig.2007.02.001

[b13] Ward R. D., Zemlak T. S., Innes B. H., Last P. R. & Hebert P. D. DNA barcoding Australia’s fish species. Philos. Trans. R Soc. Lond. B Biol. Sci. 360, 1847–1857 (2005). 16214743 10.1098/rstb.2005.1716 PMC1609232

[b14] Hebert P. D., Ratnasingham S. & deWaard J. R. Barcoding animal life: cytochrome c oxidase subunit 1 divergences among closely related species. Proc. Biol. Sci. 270 **Suppl 1**, S96–99 (2003). 12952648 10.1098/rsbl.2003.0025 PMC1698023

[b15] Kress W. J., Wurdack K. J., Zimmer E. A., Weigt L. A. & Janzen D. H. Use of DNA barcodes to identify flowering plants. Proc. Natl. Acad. Sci. USA 102, 8369–8374 (2005). 15928076 10.1073/pnas.0503123102 PMC1142120

[b16] Pang X. . Applying plant DNA barcodes for Rosaceae species identification. Cladistics 27, 165–170 (2011). 10.1111/j.1096-0031.2010.00328.x 34875771

[b17] Yao H. . Use of ITS2 region as the universal DNA barcode for plants and animals. PLoS One 5, e13102 (2010). 20957043 10.1371/journal.pone.0013102 PMC2948509

[b18] Group C. P. W. A. DNA barcode for land plants. Proc. Natl. Acad. Sci. USA 106, 12794–12797 (2009). 19666622 10.1073/pnas.0905845106 PMC2722355

[b19] Kress W. J. & Erickson D. L. A two-locus global DNA barcode for land plants: the coding *rbcL* gene complements the non-coding *trnH-psbA* spacer region. PLoS One 2, e508 (2007). 17551588 10.1371/journal.pone.0000508 PMC1876818

[b20] Chen S. . Validation of the ITS2 region as a novel DNA barcode for identifying medicinal plant species. PLoS One 5, e8613 (2010). 20062805 10.1371/journal.pone.0008613 PMC2799520

[b21] China Plant B. O. L. G. . Comparative analysis of a large dataset indicates that internal transcribed spacer (ITS) should be incorporated into the core barcode for seed plants. Proc. Natl. Acad. Sci. USA 108, 19641–19646 (2011). 22100737 10.1073/pnas.1104551108 PMC3241788

[b22] Chen S. . A renaissance in herbal medicine identification: from morphology to DNA. Biotechnol. Adv. 32, 1237–1244 (2014). 25087935 10.1016/j.biotechadv.2014.07.004

[b23] Kool A. . Molecular identification of commercialized medicinal plants in southern Morocco. PLoS One 7, e39459 (2012). 22761800 10.1371/journal.pone.0039459 PMC3384669

[b24] Chen X. . A fast SNP identification and analysis of intraspecific variation in the medicinal *Panax* species based on DNA barcoding. Gene 530, 39–43 (2013). 23933277 10.1016/j.gene.2013.07.097

[b25] Xin T. . Survey of commercial Rhodiola products revealed species diversity and potential safety issues. Sci. Rep. 5, 8337 (2015). 25661009 10.1038/srep08337 PMC4321177

[b26] Xiang L. . DNA barcoding the commercial Chinese caterpillar fungus. FEMS Microbiol. Lett. 347, 156–162 (2013). 23927075 10.1111/1574-6968.12233

[b27] Lao S. C. . Identification and quantification of 13 components in *Angelica sinensis* (Danggui) by gas chromatography–mass spectrometry coupled with pressurized liquid extraction. Analytica Chimica Acta 526, 131–137 (2004).

[b28] Shokralla S. . Next-generation DNA barcoding: using next-generation sequencing to enhance and accelerate DNA barcode capture from single specimens. Mol. Ecol. Res. 14, 892–901 (2014). 10.1111/1755-0998.12236 PMC4276293 24641208

[b29] Taberlet P., Coissac E., Pompanon F., Brochmann C. & Willerslev E. Towards next-generation biodiversity assessment using DNA metabarcoding. Mol. Ecol. 21, 2045–2050 (2012). 22486824 10.1111/j.1365-294X.2012.05470.x

[b30] Vassou S. L., Nithaniyal S., Raju B. & Parani M. Creation of reference DNA barcode library and authentication of medicinal plant raw drugs used in Ayurvedic medicine. BMC Complem. Altern. M 16 **Suppl 1**, 186, doi: 10.1186/s12906-016-1086-0 (2016). PMC4959393 27454470

[b31] Li X. & Wang H. Chinese herbal medicine in the treatment of chronic kidney disease. Advances in chronic kidney disease 12, 276–281 (2005). 16010642 10.1016/j.ackd.2005.03.007

[b32] Seeff L. B., Lindsay K. L., Bacon B. R., Kresina T. F. & Hoofnagle J. H. Complementary and alternative medicine in chronic liver disease. Hepatology 34, 595–603 (2001). 11526548 10.1053/jhep.2001.27445

[b33] Hasani-Ranjbar S., Nayebi N., Larijani B. & Abdollahi M. A systematic review of the efficacy and safety of herbal medicines used in the treatment of obesity. World J. Gastroenterol. 15, 3073–3085 (2009). 19575486 10.3748/wjg.15.3073 PMC2705729

[b34] Hasan S. S., Ahmed S. I., Bukhari N. I. & Loon W. C. W. Use of complementary and alternative medicine among patients with chronic diseases at outpatient clinics. Comp. Ther. Clin. Pract. 15, 152–157 (2009). 10.1016/j.ctcp.2009.02.003 19595416

[b35] Qi F. . Chinese herbal medicines as adjuvant treatment during chemo-or radio-therapy for cancer. Biosci. Trends 4, 297–307 (2010). 21248427

[b36] Newmaster S. G., Grguric M., Shanmughanandhan D., Ramalingam S. & Ragupathy S. DNA barcoding detects contamination and substitution in North American herbal products. BMC Med. 11, 222 (2013). 24120035 10.1186/1741-7015-11-222 PMC3851815

[b37] Mishra P. . DNA barcoding: an efficient tool to overcome authentication challenges in the herbal market. Plant Biotechnol. J. 14, 8–21 (2015). 26079154 10.1111/pbi.12419 PMC11388846

[b38] Ono T. . An accidental case of aconite poisoning due to Kampo herbal medicine ingestion. Leg. Med. (Tokyo) 11, 132–135 (2009). 19121599 10.1016/j.legalmed.2008.11.001

[b39] Chen S. P. . Aconite poisoning over 5 years: a case series in Hong Kong and lessons towards herbal safety. Drug. Saf. 35, 575–587 (2012). 22631223 10.2165/11597470-000000000-00000

[b40] Hu Z. Study on DNA barcoding and chloroplast genome of medicinal plants in Compositae. Doctoral thesis, Hubei University of Chinese Medicine (2012).

[b41] Li X. . Plant DNA barcoding: from gene to genome. Biol. Rev. Camb. Philos. Soc. 90, 157–166 (2015). 24666563 10.1111/brv.12104

[b42] Parks M., Cronn R. & Liston A. Increasing phylogenetic resolution at low taxonomic levels using massively parallel sequencing of chloroplast genomes. BMC Biol. 7, 84 (2009). 19954512 10.1186/1741-7007-7-84 PMC2793254

[b43] Chen S. L. . Principles for molecular identification of traditional Chinese materia medica using DNA barcoding. Zhongguo Zhong Yao Za Zhi 38, 141–148 (2013). 23672031

[b44] Keller A. . 5.8S-28S rRNA interaction and HMM-based ITS2 annotation. Gene 430, 50–57 (2009). 19026726 10.1016/j.gene.2008.10.012

