# Supplementary material for: Identification of crude drugs in the Japanese pharmacopoeia using a DNA barcoding system
Source: Sci Rep. 2017 Feb 10;7:42325. doi: 10.1038/srep42325 (PMC5301229; doi:10.1038/srep42325)
Supplement: Supplementary Information [file srep42325-s1.pdf]

Identification of crude drugs in the Japanese pharmacopoeia using a DNA barcoding system

Xiaochen Chen[1+], Li Xiang[2+], Linchun Shi[1], Gang Li[3], Hui Yao[1], Jianping Han[1], Yulin Lin[1], Jingyuan Song[1*], Shilin Chen[2*].

1. Institute of Medicinal Plant Development, Chinese Academy of Medical Sciences & Peking Union Medical College, Beijing 100193, P.R. China. 2. Institute of Chinese Materia Medica, China Academy of Chinese Medical Sciences, Beijing 100700, P.R. China. 3. China Medico Corporation, Talent International Building, No.80, Guangqumen Nei Street, Dongcheng District, Beijing 100062, P.R. China.

*Authors for correspondence

Shilin Chen slchen@icmm.ac.cn

Jingyuan Song jysong@implad.ac.cn

+ Authors contributing equally to this work

Table S1 Sampling and sequence information

Species Name	Sampling location	Voucher No.	Genbank No.	Region
Achyranthes bidentata	The institute of medicinal plant development,Beijing,China	PS1493MT05	GQ434786	ITS2
Achyranthes bidentata	Drugstore,Beijing,China	YC0097MT01	KX674743	ITS2
Achyranthes bidentata	Drugstore,Beijing,China	YC0097MT10	KX674744	ITS2
Achyranthes fauriei	Chengdu university of Chinese Medicine	JP045MT01	null	ITS2
Aconitum carmichaelii	The institute of medicinal plant development,Beijing,China	YC0157MT12	KX674864	ITS2
Aconitum carmichaelii	Changchun, Jilin，China	YC0157MT18	KX674929	ITS2
Aconitum carmichaelii	Chengdu university of Chinese Medicine	YC0157MT13	KX674997	ITS2
Aconitum japonicum	Chengdu university of Chinese Medicine	JP049MT01	null	ITS2
Akebia quinata	Lushan Mountain, Jiangxi，China	YC0408MT01	KX674816	ITS2
Akebia quinata	Lushan Mountain, Jiangxi，China	YC0408MT02	KX674959	ITS2
Akebia quinata	Lushan Mountain, Jiangxi，China	YC0408MT03	KX675089	ITS2
Akebia trifoliata	Lushan Mountain, Jiangxi，China	YC0409MT03	KX674894	ITS2
Akebia trifoliata	Hubei university of Chinese Medicine	YC0409MT11	KX674895	ITS2
Akebia trifoliata	Hubei university of Chinese Medicine	YC0409MT12	KX675090	ITS2
Alisma orientale	Anguo Medicinal Market,Hebei,China	YC0072MT03	KX674937	ITS2

Alisma orientale	Hehuachi Medicinal Market,Hebei,China	YC0072MT08	KX674938	ITS2
Alisma orientale	Ganzhou, Jiangxi，China	YC0072MT09	KX674939	ITS2
Aloe ferox	Fukuoka University, Fukuoka, Japan	JP001MT01	KX714227	ITS2
Aloe ferox	Fukuoka University, Fukuoka, Japan	JP001MT02	KX714228	ITS2
Aloe ferox	Fukuoka University, Fukuoka, Japan	JP001MT03	KX714229	ITS2
Alpinia officinarum	Nanning, Guangxi，China	PS0519MT01	KX674912	ITS2
Alpinia officinarum	Anguo Medicinal Market,Hebei,China	YC0220MT01	KX675031	ITS2
Alpinia officinarum	Gaungzhou University of Chinese Medicine, Guangdong，China	YC0220MT07	KX675032	ITS2
Alpinia oxyphylla	Xishuangbanna Tropical Botanical Garden, Yunnan，China	YC0103MT05	KX674890	ITS2
Alpinia oxyphylla	South China Botanical Garden，Guangdong，China	YC0103MT08	KX674891	ITS2
Alpinia oxyphylla	The institute of medicinal plant development,Beijing,China	YC0103MT10	KX675087	ITS2
Amomum villosum var. xanthioides	\	\	FJ972781	ITS2
Amomum villosum var. xanthioides	\	\	JF421467	ITS2
Amomum villosum var. xanthioides	Botanical Garden of Xishuangbanna South Medicine, Yunnan，China	PS0526MT01	KX674915	ITS2
Anemarrhena asphodeloides	The institute of medicinal plant	YC0037MT14	KX674791	ITS2

	development,Beijing,China			
Anemarrhena asphodeloides	Bozhou Medicinal Market,Anhui,China	YC0037MT08	KX674940	ITS2
Anemarrhena asphodeloides	Anguo Medicinal Market,Hebei,China	YC0037MT01	KX674941	ITS2
Angelica acutiloba	\	\	AB569093	ITS2
Angelica acutiloba	\	\	AY548227	ITS2
Angelica acutiloba	The University of Tokyo, Tokyo, Japan	JP019MT01	KU174577	ITS2
Angelica acutiloba var. sugiyamae	University of Toyama, Toyama, Japan	JP020MT01	KU174578	ITS2
Angelica acutiloba var. sugiyamae	University of Toyama, Toyama, Japan	JP020MT02	KU174579	ITS2
Angelica acutiloba var. sugiyamae	University of Toyama, Toyama, Japan	JP020MT03	KU174580	ITS2
Angelica dahurica	The institute of medicinal plant development,Beijing,China	PS1197MT01	KX674928	ITS2
Angelica dahurica	Luoyang, Henan，China	YC0152MT14	KX674998	ITS2
Angelica dahurica	Drug store, Tokyo，Japan	JPHF096	KX674999	ITS2
Angelica decursiva	Guangxi Botanical Garden of Medicinal Plants, Guizhou，China	PS1226MT05	KX674745	ITS2
Angelica decursiva	The institute of medicinal plant development,Beijing,China	PS1226MT04	KX674921	ITS2
Angelica decursiva	The institute of medicinal plant development,Beijing,China	PS1226MT04	KX674922	ITS2

Aralia cordata	The University of Tokyo, Tokyo, Japan	JP002MT01	KU174584	ITS2
Aralia cordata	University of Toyama, Toyama, Japan	JP002MT02	KU174585	ITS2
Aralia cordata	University of Toyama, Toyama, Japan	JP002MT03	KU174586	ITS2
Arctium lappa	Nanchuan, Chongqing，China	YC0252MT15	KX674764	ITS2
Arctium lappa	Bozhou Medicinal Market,Anhui,China	YC0252MT09	KX674765	ITS2
Arctium lappa	Nanyang, Henan，China	YC0252MT10	KX675016	ITS2
Arctostaphylos uva-ursi	Tsumura & Co., Japan	JP004MT01	KU174587	ITS2
Arctostaphylos uva-ursi	Tsumura & Co., Japan	JP004MT02	KU174588	ITS2
Arctostaphylos uva-ursi	Tsumura & Co., Japan	JP004MT03	KU174589	ITS2
Artemisia capillaris	Drug store, Beijing，China	YC0364MT13	KX675132	ITS2
Artemisia capillaris	Drug store, Beijing，China	YC0364MT09	KX675133	ITS2
Artemisia capillaris	National Institute for Food and Drug Control，Beijing，China	FDC369	KX675134	ITS2
Asiasarum heterotropoides var. mandshuricum	Changbaishan, Jilin，China	CBS117MT01	KX674960	ITS2
Asiasarum heterotropoides var. mandshuricum	Changchun, Jilin，China	ZL-026-02	KX674961	ITS2
Asiasarum heterotropoides var. mandshuricum	Changchun, Jilin，China	ZL-026-03	KX674962	ITS2
Asiasarum sieboldii	Wuhan, Hubei , China	YC0607MT10	KX674817	ITS2
Asiasarum sieboldii	Shaanxi Normal University, Shaanxi，China	YC0607MT01	KX674818	ITS2

Asiasarum sieboldii	Shaanxi Normal University, Shaanxi，China	YC0607MT02	KX675091	ITS2
Asparagus cochinchinensis	Guangxi Botanical Garden of Medicinal Plants, Guizhou，China	PS0057MT01	KX674819	ITS2
Asparagus cochinchinensis	Daozhen, Guizhou , China	YC0287MT01	KX674820	ITS2
Asparagus cochinchinensis	Daozhen, Guizhou , China	YC0287MT02	KX675092	ITS2
Astragalus membranaceus	The institute of medicinal plant development,Beijing,China	YC0544MT21	KX674823	ITS2
Astragalus membranaceus	Shaanxi Normal University, Shaanxi，China	YC0544MT06	KX674824	ITS2
Astragalus membranaceus	Shaanxi Normal University, Shaanxi，China	YC0544MT07	KX674963	ITS2
Astragalus mongholicus	Minxian, Gansu，China	PS0277MT01	KX674909	ITS2
Astragalus mongholicus	Minxian, Gansu，China	GS1	KJ999351	ITS2
Astragalus mongholicus	Minxian, Gansu，China	GS2	KJ999353	ITS2
Atractylodes japonica	The institute of medicinal plant development,Beijing,China	JP003MT01	KU174581	ITS2
Atractylodes japonica	The institute of medicinal plant development,Beijing,China	JP003MT02	KU174582	ITS2
Atractylodes japonica	The institute of medicinal plant development,Beijing,China	JP003MT03	KU174583	ITS2
Atractylodes lancea/Atractylodes chinensis	Anguo Medicinal Market,Hebei,China	YC0161MT08	KX674896	ITS2

Atractylodes lancea/Atractylodes chinensis	The institute of medicinal plant development,Beijing,China	YC0161MT10	KX675097	ITS2
Atractylodes lancea/Atractylodes chinensis	Nanyang, Henan，China	YC0161MT09	KX675098	ITS2
Atractylodes macrocephala	The institute of medicinal plant development,Beijing,China	YC0059MT15	KX674827	ITS2
Atractylodes macrocephala	Bozhou Medicinal Market,Anhui,China	YC0059MT08	KX674828	ITS2
Atractylodes macrocephala	Bozhou Medicinal Market,Anhui,China	YC0059MT12	KX675099	ITS2
Atropa belladonna	Nanchuan, Chongqing，China	YC0645MT04	KX674979	ITS2
Atropa belladonna	Nanchuan, Chongqing，China	YC0645MT12	KX674980	ITS2
Atropa belladonna	Nanchuan, Chongqing，China	YC0645MT13	KX674981	ITS2
Aucklandia lappa	Anguo Medicinal Market,Hebei,China	YC0139MT21	KX674792	ITS2
Aucklandia lappa	Anguo Medicinal Market,Hebei,China	YC0139MT09	KX674793	ITS2
Aucklandia lappa	Kunming, Yunnan，China	YC0139MT07	KX675056	ITS2
Benincasa cerifera	Shaanxi Normal University, Shaanxi，China	YC0136MT13	KX674774	ITS2
Benincasa cerifera	Anguo Medicinal Market,Hebei,China	YC0136MT05	KX674775	ITS2
Benincasa cerifera	Chuqimen Medicinal Market, Chongqing，China	YC0136MT07	KX674934	ITS2
Bupleurum falcatum	Fukuoka University, Fukuoka, Japan	JP006MT01	KU174605	ITS2
Bupleurum falcatum	Fukuoka University, Fukuoka, Japan	JP006MT02	KU174606	ITS2

Bupleurum falcatum	Fukuoka University, Fukuoka, Japan	JP006MT03	KU174607	ITS2
Caesalpinia sappan	Xishuangbanna, Yunnan，China	YC0267MT08	KX674882	ITS2
Caesalpinia sappan	National Institute for Food and Drug Control，Beijing，China	YC0267MT05	KX674883	ITS2
Caesalpinia sappan	Drug store, Beijing, China	YC0267MT09	KX675036	ITS2
Cannabis sativa	The institute of medicinal plant development,Beijing,China	PS0216MT01	KX674776	ITS2
Cannabis sativa	Yulin Medicinal Market,Gaungxi,China	YC0259MT08	KX674777	ITS2
Cannabis sativa	Drug store,Beijing,China	YC0259MT09	KX674908	ITS2
Capsicum annuum	Guangxi Botanical Garden of Medicinal Plants, Guizhou，China	YC0378MT07	KX674794	ITS2
Capsicum annuum	Anguo Medicinal Market,Hebei,China	YC0378MT09	KX674942	ITS2
Capsicum annuum	Changsha, Hunan, China	YC0378MT04	KX674943	ITS2
Carthamus tinctorius	Anguo Medicinal Market,Hebei,China	YC0013MT08	KX674843	ITS2
Carthamus tinctorius	Yunlin, Guangxi,China	YC0013MT02	KX674982	ITS2
Carthamus tinctorius	Anguo Medicinal Market,Hebei,China	YC0013MT12	KX675122	ITS2
Cassia acutifolia	\	\	JQ301846	ITS2
Cassia acutifolia	\	\	KF815491	ITS2
Cassia angustifolia	Tsumura & Co., Japan	JP040MT01	KU174617	ITS2

Cassia angustifolia	Tsumura & Co., Japan	JP040MT02	KU174618	ITS2
Cassia angustifolia	Tsumura & Co., Japan	JP040MT03	KU174619	ITS2
Cassia obtusifolia	Nanning, Guangxi， China	PS1588MT01	KX674795	ITS2
Cassia obtusifolia	Nanyang, Henan， China	YC0048MT08	KX674796	ITS2
Cassia obtusifolia	Nanyang, Henan， China	YC0048MT10	KX675057	ITS2
Cassia tora	Anguo Medicinal Market,Hebei,China	YC0113MT11	KX674797	ITS2
Cassia tora	Nanyang, Henan， China	YC0113MT06	KX674798	ITS2
Cassia tora	Nanyang, Henan， China	YC0113MT08	KX674944	ITS2
Catalpa bungei	Beijing Botanical Garden, Beijing, China	JP008MT01	KU174634	ITS2
Catalpa bungei	Beijing Botanical Garden, Beijing, China	JP008MT02	KU174635	ITS2
Catalpa bungei	Beijing Botanical Garden, Beijing, China	JP008MT03	KU174636	ITS2
Catalpa ovata	Beijing Botanical Garden, Beijing, China	JP007MT01	KU174629	ITS2
Catalpa ovata	Nanchuan, Chongqing, China	JP007MT04	KU174630	ITS2
Catalpa ovata	Nanchuan, Chongqing, China	JP007MT05	KU174631	ITS2
Cephaelis ipecacuanha/Cephaelis acuminata	Tsumura & Co., Japan	JP018MT01	KU174574	ITS2
Cephaelis ipecacuanha/Cephaelis acuminata	Tsumura & Co., Japan	JP018MT02	KU174575	ITS2
Cephaelis ipecacuanha/Cephaelis acuminata	Tsumura & Co., Japan	JP018MT03	KU174576	ITS2
Chrysanthemum indicum	The institute of medicinal plant development,Beijing,China	YC0100MT34	KX675017	ITS2

Chrysanthemum indicum	Drug store, Beijing, China	YC0100MT29	KX675018	ITS2
Chrysanthemum indicum	National Institute for Food and Drug Control，Beijing，China	YC0100MT30	KX675019	ITS2
Chrysanthemum morifolium	The institute of medicinal plant development,Beijing,China	YC0005MT51	KX674766	ITS2
Chrysanthemum morifolium	Bozhou, Anhui，China	YC0005MT26	KX674767	ITS2
Chrysanthemum morifolium	Jiaozuo, Henan，China	YC0005MT29	KX675020	ITS2
Cimicifuga dahurica	Changbaishan, Jilin，China	YC0500MT02	KX674804	ITS2
Cimicifuga dahurica	Bozhou, Anhui，China	YC0500MT06	KX674805	ITS2
Cimicifuga dahurica	Bozhou, Anhui，China	YC0500MT07	KX674806	ITS2
Cimicifuga foetida	Bozhou Medicinal Market,Anhui,China	YC0335MT02	KX675064	ITS2
Cimicifuga foetida	Nanchuan, Chongqing, China	YC0060MT01	KX675065	ITS2
Cimicifuga foetida	Haerbin, Heilongjiang , China	YC0060MT09	KX675066	ITS2
Cimicifuga simplex	Hehuachi Medicinal Market,Hebei,China	YC0060MT10	KJ487991	ITS2
Cimicifuga simplex	Hehuachi Medicinal Market,Hebei,China	YC0592MT07	KJ487992	ITS2
Cimicifuga simplex	Haerbin, Heilongjiang，China	YC0592MT08	KJ487993	ITS2
Cimisifuga heracleifolia	Changbaishan, Jilin , China	YC0335MT10	KX675067	ITS2
Cimisifuga heracleifolia	Drug store, Beijing, China	YC0335MT13	KX675068	ITS2
Cimisifuga heracleifolia	Changbaishan, Jilin , China	YC0335MT05	KX675069	ITS2

Citrus aurantium	Nanchuan, Chongqing，China	YC0145MT29	KX674889	ITS2
Citrus aurantium	Nanchuan, Chongqing，China	YC0145MT21	KX675070	ITS2
Citrus aurantium	Anguo Medicinal Market,Hebei,China	YC0145MT22	KX675071	ITS2
Citrus aurantium var. daidai	Fukuoka University, Fukuoka, Japan	JP005MT01	KU174590	ITS2
Citrus aurantium var. daidai	Fukuoka University, Fukuoka, Japan	JP005MT02	KU174591	ITS2
Citrus aurantium var. daidai	Fukuoka University, Fukuoka, Japan	JP005MT03	KU174592	ITS2
Citrus natsudaidai	Tsumura & Co., Japan	JP017MT01	KU174596	ITS2
Citrus natsudaidai	Tsumura & Co., Japan	JP017MT02	KU174597	ITS2
Citrus natsudaidai	Tsumura & Co., Japan	JP017MT03	KU174598	ITS2
Citrus reticulata	Drug store, Beijing, China	YC0012MT22	KX674884	ITS2
Citrus reticulata	Drug store, Beijing, China	YC0012MT08	KX674885	ITS2
Citrus reticulata	Bozhou Medicinal Market,Anhui,China	YC0012MT15	KX675072	ITS2
Citrus unshiu	Zhejiang A & F University, Zhejiang, China	JP010MT01	KU174593	ITS2
Citrus unshiu	Zhejiang A & F University, Zhejiang, China	JP010MT02	KU174594	ITS2
Citrus unshiu	Zhejiang A & F University, Zhejiang, China	JP010MT03	KU174595	ITS2
Clematis chinensis	Drug store, Tokyo, Japan	JPHF041	KX674954	ITS2
Clematis chinensis	The institute of medicinal plant development,Beijing,China	YC0630MT09	KX674955	ITS2
Clematis chinensis	Changbaishan, Jilin，China	YC0630MT04	KX674956	ITS2

Clematis hexapetala	Changbaishan, Jilin，China	CBS221MT02	KX674807	ITS2
Clematis hexapetala	Changbaishan, Jilin，China	YC0485MT01	KX674865	ITS2
Clematis hexapetala	Drug store, Beijing, China	YC0485MT06	KX674953	ITS2
Clematis mandshurica	Anguo Medicinal Market,Anhui,China	YC0470MT05	KX674886	ITS2
Clematis mandshurica	Bozhou Medicinal Market,Anhui,China	YC0470MT06	KX674887	ITS2
Clematis mandshurica	Bozhou Medicinal Market,Anhui,China	YC0470MT01	KX674888	ITS2
Cnidium monnieri	Nanning, Guangxi，China	PS1219MT01	KX674772	ITS2
Cnidium monnieri	Anguo Medicinal Market,Hebei,China	YC0049MT04	KX674773	ITS2
Cnidium monnieri	Anguo Medicinal Market,Hebei,China	YC0049MT05	KX675029	ITS2
Cnidium officinale	\	\	JN853780	ITS2
Cnidium officinale	\	\	KJ025063	ITS2
Cnidium officinale	\	\	U78448	ITS2
Coix lacryma-jobi var. mayuen	The institute of medicinal plant development,Beijing,China	YC0204MT12	KX674857	ITS2
Coix lacryma-jobi var. mayuen	Chuqimen Medicinal Market,Chongqing,China	YC0204MT06	KX674858	ITS2
Coix lacryma-jobi var. mayuen	Xinhehuachi Medicinal Market,Sichuan,China	YC0204MT03	KX675135	ITS2
Coptis chinensis	Sichuan University, Sichuan，China	PS0915MT02	KX674799	ITS2
Coptis chinensis	Bozhou Medicinal Market,Anhui,China	YC0126MT15	KX674800	ITS2

Coptis chinensis	Bozhou Medicinal Market,Anhui,China	YC0126MT12	KX674917	ITS2
Coptis deltoidea	Botanical Garden of Xishuangbanna South Medicine, Yunnan，China	PS0924MT01	KX674918	ITS2
Coptis deltoidea	Sichuan University, Sichuan，China	YC0489MT05	KX675058	ITS2
Coptis deltoidea	Sichuan University, Sichuan，China	YC0489MT02	KX675059	ITS2
Coptis japonica	Tsumura & Co., Japan	JP012MT01	KU174541	ITS2
Coptis japonica	Tsumura & Co., Japan	JP012MT02	KU174542	ITS2
Coptis japonica	University of Toyama, Toyama, Japan	JP012MT03	KU174543	ITS2
Coptis teeta	Botanical Garden of Xishuangbanna South Medicine, Yunnan，China	YC0380MT06	KX674801	ITS2
Coptis teeta	Botanical Garden of Xishuangbanna South Medicine, Yunnan，China	YC0380MT01	KX675060	ITS2
Coptis teeta	Botanical Garden of Xishuangbanna South Medicine, Yunnan，China	YC0380MT02	KX675061	ITS2
Cornus officinalis	Drug store, Beijing, China	YC0076MT23	KX674739	ITS2
Cornus officinalis	Drug store, Beijing, China	YC0076MT24	KX674740	ITS2
Cornus officinalis	Nanyang, Henan，China	YC0076MT31	KX674741	ITS2
Corydalis yanhusuo	Nanyang, Henan，China	YC0121MT10	KX674988	ITS2
Corydalis yanhusuo	Yulin Medicinal Market,Gaungxi,China	YC0121MT05	KX674989	ITS2

Corydalis yanhusuo	Bozhou Medicinal Market,Anhui,China	YC0121MT06	KX674990	ITS2
Crataegus pinnatifida var. major	The institute of medicinal plant development,Beijing,China	YC0193MT10	KX674964	ITS2
Crataegus pinnatifida var. major	National Institute for Food and Drug Control，Beijing，China	FDC240	KX674965	ITS2
Crataegus pinnatifida var. major	Nanyang, Henan，China	YC0193MT01	KX675094	ITS2
Crocus sativus	Anguo Medicinal Market,Hebei,China	YC0232MT05	KX674983	ITS2
Crocus sativus	Anguo Medicinal Market,Hebei,China	YC0232MT06	KX674984	ITS2
Crocus sativus	Bozhou Medicinal Market,Anhui,China	YC0232MT07	KX675123	ITS2
Curcuma longa	Nanning, Guangxi,China	PS0525MT03	KX674742	ITS2
Curcuma longa	Nanning, Guangxi,China	YC0127MT07	KX675000	ITS2
Curcuma longa	Nanning, Guangxi,China	YC0127MT08	KX675001	ITS2
Curcuma zedoaria	Nanning, Guangxi,China	PS0518MT04	KX674746	ITS2
Cyperus rotundus	Ganzhou, Jiangxi，China	YC0142MT07	KX674892	ITS2
Cyperus rotundus	Anguo Medicinal Market,Hebei,China	YC0142MT10	KX674893	ITS2
Cyperus rotundus	Anguo Medicinal Market,Hebei,China	YC0142MT11	KX675088	ITS2
Digenea simplex	Tsumura & Co., Japan	JP048MT01	null	ITS2
Digenea simplex	Tsumura & Co., Japan	JP048MT02	null	ITS2
Digenea simplex	Tsumura & Co., Japan	JP048MT03	null	ITS2

Dimocarpus longan	Putian, Fujian，China	PS1439MT04	KX674747	ITS2
Dimocarpus longan	Drug store, Beijing, China	YC0576MT06	KX674748	ITS2
Dimocarpus longan	Drug store, Beijing, China	YC0576MT07	KX675002	ITS2
Dolichos lablab	Anguo Medicinal Market,Hebei,China	YC0242MT15	KX674859	ITS2
Dolichos lablab	Anguo Medicinal Market,Hebei,China	YC0242MT05	KX674860	ITS2
Dolichos lablab	Bozhou Medicinal Market,Anhui,China	YC0242MT06	KX675136	ITS2
Elettaria cardamomum	Tsumura & Co., Japan	JP047MT01	null	ITS2
Elettaria cardamomum	Tsumura & Co., Japan	JP047MT02	null	ITS2
Elettaria cardamomum	Tsumura & Co., Japan	JP047MT03	null	ITS2
Eleutherococcus senticosus	The institute of medicinal plant development,Beijing,China	YC0465MT17	KX674737	ITS2
Eleutherococcus senticosus	Anguo Medicinal Market,Hebei,China	YC0465MT08	KX674738	ITS2
Eleutherococcus senticosus	Bozhou Medicinal Market,Anhui,China	YC0465MT11	KX674996	ITS2
Ephedra equisetina	Hetian, Xinjiang，China	YC0708MT21	KX675037	ITS2
Ephedra equisetina	The institute of medicinal plant development,Beijing,China	YC0708MT15	KX675038	ITS2
Ephedra equisetina	The institute of medicinal plant development,Beijing,China	YC0708MT16	KX675039	ITS2
Ephedra intermedia	Hetian, Xinjiang，China	YC0347MT32	KX675040	ITS2

Ephedra intermedia	Hetian, Xinjiang，China	YC0347MT23	KX675041	ITS2
Ephedra intermedia	Hetian, Xinjiang，China	YC0347MT22	KX675042	ITS2
Ephedra sinica	Chifeng, Neimenggu，China	YC0118MT12	KX675043	ITS2
Ephedra sinica	Datong, Shanxi, China	YC0118MT02	KX675044	ITS2
Ephedra sinica	Chifeng, Neimenggu，China	YC0118MT03	KX675045	ITS2
Epimedium brevicornu	Shangluo, Shaanxi，China	YC0626MT09	KX674949	ITS2
Epimedium brevicornu	National Institute for Food and Drug Control，Beijing，China	FDC205	KX675073	ITS2
Epimedium brevicornu	National Institute for Food and Drug Control，Beijing，China	FDC206	KX675074	ITS2
Epimedium grandiflorum var. thunbergianum	The University of Tokyo, Tokyo, Japan	JP013MT01	KU174637	ITS2
Epimedium grandiflorum var. thunbergianum	Tokyo, Japan	JP013MT02	KU174638	ITS2
Epimedium grandiflorum var. thunbergianum	University of Toyama, Toyama, Japan	JP013MT03	KU174639	ITS2
Epimedium koreanum	Xinbin, Liaoning，China	YC0464MT05	KX674951	ITS2
Epimedium koreanum	Xinbin, Liaoning，China	YC0464MT07	KX675075	ITS2
Epimedium koreanum	Xinbin, Liaoning，China	YC0464MT08	KX675076	ITS2
Epimedium pubescens	Chenshan Botanical Gaden, Shanghai，China	YC0440MT08	KX674866	ITS2
Epimedium pubescens	Danfeng, Shaanxi，China	PS1505MT01	KX675077	ITS2
Epimedium pubescens	Nanchuan, Chongqing, China	YC0440MT02	KX675078	ITS2

Epimedium sagittatum	Chengdu, Sichuan，China	YC0441MT06	KX674808	ITS2
Epimedium sagittatum	The institute of medicinal plant development,Beijing,China	PS1502MT02	KX675079	ITS2
Epimedium sagittatum	Chengdu, Sichuan，China	YC0441MT07	KX675080	ITS2
Epimedium sempervirens	University of Toyama, Toyama, Japan	JP014MT01	KU174640	ITS2
Epimedium sempervirens	University of Toyama, Toyama, Japan	JP014MT02	KU174641	ITS2
Epimedium sempervirens	University of Toyama, Toyama, Japan	JP014MT03	KU174642	ITS2
Epimedium wushanense	Chenshan Botanical Gaden, Shanghai，China	YC0442MT07	KX674867	ITS2
Epimedium wushanense	Shennongjia, Hubei，China	YC0442MT14	KX674952	ITS2
Epimedium wushanense	Chengdu, Sichuan，China	YC0442MT04	KX675081	ITS2
Eriobotrya japonica	National Institute for Food and Drug Control，Beijing，China	FDC199	KX674809	ITS2
Eriobotrya japonica	Bozhou Medicinal Market,Anhui,China	YC0217MT04	KX674950	ITS2
Eriobotrya japonica	Bozhou Medicinal Market,Anhui,China	YC0217MT08	KX675082	ITS2
Eucommia ulmoides	The institute of medicinal plant development,Beijing,China	YC0091MT21	KX674935	ITS2
Eucommia ulmoides	The institute of medicinal plant development,Beijing,China	YC0091MT22	KX674936	ITS2
Eucommia ulmoides	Bozhou Medicinal Market,Anhui,China	YC0091MT03	KX675052	ITS2

Eugenia caryophyllata	Botanical Garden of Xishuangbanna South Medicine, Yunnan，China	YC0224MT11	KX675083	ITS2
Eugenia caryophyllata	Botanical Garden of Xishuangbanna South Medicine, Yunnan，China	YC0224MT12	KX675084	ITS2
Eugenia caryophyllata	Botanical Garden of Xishuangbanna South Medicine, Yunnan，China	YC0224MT13	KX675085	ITS2
Euodia ruticarpa/Euodia officinalis/Euodia bodinieri	Nanchuan, Chongqing, China	YC0276MT28	KX674957	ITS2
Euodia ruticarpa/Euodia officinalis/Euodia bodinieri	Bozhou Medicinal Market,Anhui,China	YC0276MT32	KX674958	ITS2
Euodia ruticarpa/Euodia officinalis/Euodia bodinieri	Bozhou Medicinal Market,Anhui,China	YC0276MT31	KX675030	ITS2
Foeniculum vulgare	Shennongjia, Hubei，China	YC0169MT15	KX674861	ITS2
Foeniculum vulgare	Bozhou Medicinal Market,Anhui,China	YC0169MT02	KX674862	ITS2
Foeniculum vulgare	Anguo Medicinal Market,Hebei,China	YC0169MT06	KX674991	ITS2
Forsythia suspensa	Shennongjia, Hubei，China	YC0058MT17	KX674846	ITS2
Forsythia suspensa	Bozhou Medicinal Market,Anhui,China	YC0058MT12	KX674847	ITS2
Forsythia suspensa	Bozhou Medicinal Market,Anhui,China	YC0058MT14	KX674985	ITS2
Forsythia viridissima	Fujian Agriculture and Forestry University，Fujian, China	JP015MT01	KU174568	ITS2
Forsythia viridissima	Fujian Agriculture and Forestry University，Fujian,	JP015MT02	KU174569	ITS2

	China			
Forsythia viridissima	Chengdu, Sichuan，China	JP015MT03	KU174570	ITS2
Fritillaria thunbergii	Hetian, Xinjiang，China	YC0132MT14	KX674829	ITS2
Fritillaria thunbergii	Yinxian, Zhejiang, China	YC0132MT24	KX674978	ITS2
Fritillaria thunbergii	Yinxian, Zhejiang, China	YC0132MT35	KX675100	ITS2
Gardenia jasminoides	Chuqimen Medicinal Market,Chongqing,China	YC0032MT13	KX675046	ITS2
Gardenia jasminoides	Anguo Medicinal Market,Hebei,China	YC0032MT09	KX675047	ITS2
Gardenia jasminoides	Shennongjia, Hubei，China	YC0032MT21	KX675048	ITS2
Gastrodia elata	Nanchuan, Chongqing, China	YC0015MT07	KX674868	ITS2
Gastrodia elata	Nanchuan, Chongqing, China	YC0015MT11	KX674869	ITS2
Gastrodia elata	Nanchuan, Chongqing, China	YC0015MT12	KX674870	ITS2
Gentiana lutea	\	\	DQ358878	ITS2
Gentiana lutea	\	\	KC535855	ITS2
Gentiana manshurica	Heilongjiang，China	YC0643MT07	KX675109	ITS2
Gentiana manshurica	Heilongjiang，China	YC0643MT02	KX675110	ITS2
Gentiana manshurica	Heilongjiang，China	YC0643MT03	KX675111	ITS2
Gentiana scabra	Liaoning，China	YC0131MT09	KX675112	ITS2
Gentiana scabra	Heilongjiang，China	YC0131MT06	KX675113	ITS2
Gentiana scabra	National Institute for Food and Drug Control，	FDC283	KX675114	ITS2

	Beijing，China			
Gentiana triflora	Gannan, Heilongjiang，China	PS2562MT01	KX674873	ITS2
Gentiana triflora	Lindian, Heilongjiang，China	YC0644MT01	KX675115	ITS2
Gentiana triflora	Lindian, Heilongjiang，China	YC0644MT02	KX675116	ITS2
Geranium thunbergii	University of Toyama, Toyama, Japan	JP016MT03	KU174620	ITS2
Geranium thunbergii	Drug store, Tokyo, Japan	JP016MT04	KU174621	ITS2
Geranium thunbergii	Tsumura & Co., Japan	JP016MT05	KU174622	ITS2
Glehnia littoralis	Bozhou Medicinal Market,Anhui,China	YC0153MT04	KX674780	ITS2
Glehnia littoralis	Anguo Medicinal Market,Hebei,China	YC0153MT05	KX674781	ITS2
Glehnia littoralis	National Institute for Food and Drug Control，Beijing，China	YC0153MT06	KX674782	ITS2
Glycyrrhiza glabra	Shihezi, Xinjiang，China	YC0399MT18	KX674930	ITS2
Glycyrrhiza glabra	Hejingxian, Xinjiang，China	RCYC0399MT01	KX675021	ITS2
Glycyrrhiza glabra	Hejingxian, Xinjiang，China	YC0399MT01	KX675022	ITS2
Glycyrrhiza uralensis	Drugstore , Beijing , China	YC0130MT37	KX674768	ITS2
Glycyrrhiza uralensis	Datong, Shanxi，China	YC0130MT20	KX674769	ITS2
Glycyrrhiza uralensis	Daqing, Heilongjiang，China	YC0130MT21	KX675023	ITS2
Houttuynia cordata	Nanchuan, Chongqing, China	PS1180MT11	KX674835	ITS2
Houttuynia cordata	Sanquan, Chongqing, China	YC0214MT10	KX674836	ITS2

Houttuynia cordata	Sanquan, Chongqing, China	YC0214MT11	KX674837	ITS2
Hydrangea macrophylla var. thunbergii	The University of Tokyo, Tokyo, Japan	JP041MT01	KU174599	ITS2
Hydrangea macrophylla var. thunbergii	The University of Tokyo, Tokyo, Japan	JP041MT02	KU174600	ITS2
Hydrangea macrophylla var. thunbergii	The University of Tokyo, Tokyo, Japan	JP041MT03	KU174601	ITS2
Imperata cylindrica var.major	The institute of medicinal plant development,Beijing,China	YC0052MT13	KX674905	ITS2
Imperata cylindrica var.major	Xinhehuachi Medicinal Market,Sichuan,China	YC0052MT08	KX674906	ITS2
Imperata cylindrica var.major	Chuqimen Medicinal Market,Chongqing,China	YC0052MT05	KX675131	ITS2
Jateorhiza columba	Tsumura & Co., Japan	JP046MT01	null	ITS2
Jateorhiza columba	Tsumura & Co., Japan	JP046MT02	null	ITS2
Jateorhiza columba	Tsumura & Co., Japan	JP046MT03	null	ITS2
Leonurus japonicus	Nanning, Guangxi， China	PS0154MT02	KX675124	ITS2
Leonurus japonicus	Nanchuan, Chongqing, China	YC0038MT07	KX675125	ITS2
Leonurus japonicus	Anguo Medicinal Market,Hebei,China	YC0038MT05	KX675126	ITS2
Leonurus sibiricus	The institute of medicinal plant development,Beijing,China	JP022MT01	KU174557	ITS2
Leonurus sibiricus	Beijing Batanical Garden, Beijing, China	JP022MT02	KU174651	ITS2
Lilium brownii	Nanchuan, Chongqing, China	JP023MT01	KU174626	ITS2

Lilium brownii	Nanchuan, Chongqing, China	JP023MT02	KU174627	ITS2
Lilium brownii	Nanchuan, Chongqing, China	JP023MT03	KU174628	ITS2
Lilium brownii var. viridulum	Shennongjia, Hubei，China	YC0747MT05	KX674830	ITS2
Lilium brownii var. viridulum	Lushan Mountain, Jiangxi，China	YC0747MT01	KX674831	ITS2
Lilium brownii var. viridulum	Lushan Mountain, Jiangxi，China	YC0747MT02	KX674970	ITS2
Lilium lancifolium	Lushan Mountain, Jiangxi，China	YC0746MT09	KX674832	ITS2
Lilium lancifolium	Bozhou Medicinal Market,Anhui,China	YC0746MT02	KX674833	ITS2
Lilium lancifolium	Mudanjiang, Heilongjiang，China	YC0746MT05	KX675101	ITS2
Lilium pumilum	Qingling, Shaanxi，China	PS0064MT02	KX674834	ITS2
Lilium pumilum	Drugstore , Beijing , China	YC0780MT02	KX674971	ITS2
Lilium pumilum	Drugstore , Beijing , China	YC0780MT02	KX675102	ITS2
Lithospermum erythrorhizon	University of Toyama, Toyama, Japan	JP024MT01	KU174571	ITS2
Lithospermum erythrorhizon	University of Toyama, Toyama, Japan	JP024MT02	KU174572	ITS2
Lithospermum erythrorhizon	The University of Tokyo, Tokyo, Japan	JPHF111	KU174573	ITS2
Lonicera japonica	Nanchuan, Chongqing, China	YC0014MT132	KX674749	ITS2
Lonicera japonica	Drugstore , Beijing , China	YC0014MT139	KX674750	ITS2
Lonicera japonica	Drugstore , Beijing , China	YC0014MT134	KX675003	ITS2
Lycium barbarum	The institute of medicinal plant development,Beijing,China	PS1800MT35	KX674901	ITS2

Lycium barbarum	Drugstore , Beijing , China	YC0026MT07	KX674902	ITS2
Lycium barbarum	Drugstore , Beijing , China	YC0026MT08	KX675117	ITS2
Lycium chinense	The institute of medicinal plant development,Beijing,China	PS1139MT20	KX674903	ITS2
Lycium chinense	Baoding, Hebei , China	PS1139MT15	KX674904	ITS2
Lycium chinense	Baoding, Hebei , China	PS1139MT16	KX675118	ITS2
Mallotus japonicus	zhengjiang A&F University, Zhejiang, China	JP027MT01	KU174558	ITS2
Marsdenia cundurango	Tsumura & Co., Japan	JP011MT01	KU174649	ITS2
Marsdenia cundurango	Tsumura & Co., Japan	JP011MT02	KU174650	ITS2
Mentha arvensis var. piperascens	Fukuoka University, Fukuoka, Japan	JP028MT01	KU174623	ITS2
Mentha arvensis var. piperascens	Fukuoka University, Fukuoka, Japan	JP028MT02	KU174624	ITS2
Mentha arvensis var. piperascens	Fukuoka University, Fukuoka, Japan	JP028MT03	KU174625	ITS2
Morus alba	Lushan, Jiangsu，China	YC0074MT37	KX674802	ITS2
Morus alba	Anguo Medicinal Market,Hebei,China	YC0074MT17	KX674803	ITS2
Morus alba	Nanning, Guangxi,China	YC0074MT18	KX675062	ITS2
Nelumbo nucifera	National Institute for Food and Drug Control，Beijing，China	FDC129	KX674783	ITS2
Nelumbo nucifera	Drugstore , Beijing , China	YC0041MT27	KX674784	ITS2
Nelumbo nucifera	Drugstore , Beijing , China	YC0041MT26	KX675053	ITS2

Notopterygium forbesii	\	\	JF755944	ITS2
Notopterygium forbesii	Xining, Qinghai,China	Q13	KX674898	ITS2
Notopterygium forbesii	Lanzhou, Gansu, China	Q15	KX675119	ITS2
Notopterygium incisum	\	\	JF755942	ITS2
Notopterygium incisum	Xining, Qinghai,China	YC0137MT06	KX674900	ITS2
Notopterygium incisum	Lanzhou, Gansu, China	YC0137MT07	KX675120	ITS2
Nuphar japonicum	Beijing Batanical Garden, Beijing, China	JP029MT04	KU174643	ITS2
Nuphar japonicum	Chenshan Botanical Gaden, Shanghai , China	JP029MT05	KU174644	ITS2
Nuphar japonicum	University of Toyama, Toyama, Japan	JP029MT03	KU174645	ITS2
Ophiopogon japonicus	Sanmen, Zhejiang, China	YC0020MT104	KX674785	ITS2
Ophiopogon japonicus	Sanmen, Zhejiang, China	YC0020MT105	KX674786	ITS2
Ophiopogon japonicus	Sanmen, Zhejiang, China	YC0020MT106	KX675049	ITS2
Oryza sativa	Drugstore，Beijing，China	YC0400MT08	KX675024	ITS2
Oryza sativa	Drugstore，Zhejiang，China	YC0400MT09	KX675025	ITS2
Oryza sativa	Chengdu, Sichuan，China	YC0400MT15	KX675026	ITS2
Paeonia lactiflora	Anguo Medicinal Market,Hebei,China	YC0101MT20	KX674931	ITS2
Paeonia lactiflora	National Institute for Food and Drug Control，Beijing，China	FDC036	KX674932	ITS2
Paeonia lactiflora	Changbaishan, Jilin，China	YC0101MT13	KX675027	ITS2

Paeonia suffruticosa	Nanchuan, Chongqing, China	YC0061MT11	KX674825	ITS2
Paeonia suffruticosa	Fangxian, Hebei，China	YC0061MT13	KX674826	ITS2
Paeonia suffruticosa	Anguo Medicinal Market,Hebei,China	YC0061MT22	KX675095	ITS2
Panax ginseng	Changbaishan, Jilin，China	YC0031MT08	KX674875	ITS2
Panax ginseng	Drugstore,Shanghai,China	YC0031MT18	KX674876	ITS2
Panax ginseng	Drugstore,Shanghai,China	YC0031MT19	KX674877	ITS2
Panax japonicus	The University of Tokyo, Tokyo, Japan	PS1477MT04	KX674924	ITS2
Panax japonicus	The University of Tokyo, Tokyo, Japan	PS1477MT02	KX674925	ITS2
Panax japonicus	The University of Tokyo, Tokyo, Japan	PS1477MT03	KX674926	ITS2
Perilla frutescens var. acuta	Fujian Agriculture and Forestry University , Fujian, China	JP030MT01	KU174608	ITS2
Perilla frutescens var. acuta	Fujian Agriculture and Forestry University , Fujian, China	JP030MT02	KU174609	ITS2
Perilla frutescens var. acuta	Fujian Agriculture and Forestry University , Fujian, China	JP030MT03	KU174610	ITS2
Perilla frutescens var. crispa	University of Toyama, Toyama, Japan	JP030MT04	KU174611	ITS2
Perilla frutescens var. crispa	University of Toyama, Toyama, Japan	JP030MT05	KU174612	ITS2
Perilla frutescens var. crispa	University of Toyama, Toyama, Japan	JP030MT06	KU174613	ITS2
Peucedanum praeruptorum	Bijie, Guizhou，China	YC0574MT03	KX674751	ITS2

Peucedanum praeruptorum	Drugstore , Beijing , China	YC0574MT05	KX674752	ITS2
Peucedanum praeruptorum	Drugstore , Beijing , China	YC0574MT06	KX675004	ITS2
Pharbitis nil	Drugstore , Beijing , China	YC0567MT13	KX674770	ITS2
Pharbitis nil	Drugstore , Beijing , China	YC0567MT04	KX674771	ITS2
Pharbitis nil	Shennongjia, Hubei , China	YC0567MT05	KX675028	ITS2
Phellodendron amurense	Changbaishan, Jilin，China	CBS478MT01	KX674848	ITS2
Phellodendron amurense	The institute of medicinal plant development,Beijing,China	YC0068MT06	KX674849	ITS2
Phellodendron amurense	The institute of medicinal plant development,Beijing,China	YC0068MT07	KX674987	ITS2
Phellodendron chinense	Shennongjia, Hubei , China	YC0079MT25	KX674850	ITS2
Phellodendron chinense	Xinhehuachi Medicinal Market,Sichuan,China	YC0079MT20	KX674851	ITS2
Phellodendron chinense	Chuqimen Medicinal Market,Chongqing,China	YC0079MT12	KX674986	ITS2
Picrasma quassioides	Pengzhou, Sichuan，China	PS0751MT01	KX674880	ITS2
Picrasma quassioides	Bozhou Medicinal Market,Anhui,China	YC0272MT02	KX674881	ITS2
Picrasma quassioides	Bozhou Medicinal Market,Anhui,China	YC0272MT02	KX674916	ITS2
Pinellia ternata	Nanyang, Henan，China	YC0023MT03	KX674787	ITS2
Pinellia ternata	The institute of medicinal plant development,Beijing,China	YC0023MT09	KX674788	ITS2

Pinellia ternata	The institute of medicinal plant development,Beijing,China	YC0023MT10	KX675050	ITS2
Pinus massoniana	Bozhou Medicinal Market,Anhui,China	YC0404MT05	KX674852	ITS2
Pinus massoniana	Bozhou Medicinal Market,Anhui,China	YC0404MT06	KX674853	ITS2
Pinus massoniana	Bozhou Medicinal Market,Anhui,China	YC0404MT07	KX674854	ITS2
Plantago asiatica	Xinglong Tropical Medicinal Botanical Garden，Hainan,China	YC0426MT13	KX674789	ITS2
Plantago asiatica	Bozhou Medicinal Market,Anhui,China	YC0426MT30	KX674790	ITS2
Plantago asiatica	Bozhou Medicinal Market,Anhui,China	YC0426MT31	KX675051	ITS2
Platycodon grandiflorum	Nanchuan, Chongqing, China	YC0016MT14	KX674838	ITS2
Platycodon grandiflorum	Nanchuan, Chongqing, China	YC0016MT15	KX674839	ITS2
Platycodon grandiflorum	Nanchuan, Chongqing, China	YC0016MT16	KX674840	ITS2
Pogostemon cablin	Guangzhou, Guangdong，China	YC0099MT03	KX674874	ITS2
Pogostemon cablin	Guangzhou, Guangdong，China	YC0099MT04	KX675127	ITS2
Pogostemon cablin	Anguo Medicinal Market,Hebei,China	YC0099MT05	KX675128	ITS2
Polygala senega	Tsumura & Co., Japan	JP038MT01	KU174559	ITS2
Polygala senega	Tsumura & Co., Japan	JP038MT02	KU174560	ITS2
Polygala senega	Tsumura & Co., Japan	JP038MT03	KU174561	ITS2

Polygala senega var. latifolia	Tsumura & Co., Japan	JP039MT01	KU174562	ITS2
Polygala senega var. latifolia	Tsumura & Co., Japan	JP039MT02	KU174563	ITS2
Polygala senega var. latifolia	Tsumura & Co., Japan	JP039MT03	KU174564	ITS2
Polygala tenuifolia	Changbaishan, Jilin， China	YC0065MT01	KX674947	ITS2
Polygala tenuifolia	Shaanxi Normal University, Shaanxi , China	YC0065MT13	KX674948	ITS2
Polygala tenuifolia	Datong, Shanxi， China	YC0065MT14	KX675086	ITS2
Polygonum multiflorum	Nanyang, Henan， China	YC0175MT20	KX675103	ITS2
Polygonum multiflorum	Nanyang, Henan， China	YC0175MT21	KX675104	ITS2
Polygonum multiflorum	Nanyang, Henan， China	YC0175MT22	KX675105	ITS2
Polyporus umbellatus	Bozhou Medicinal Market,Anhui,China	PS0338MT01	KX674910	ITS2
Polyporus umbellatus	Anguo Medicinal Market,Hebei,China	YC0018MT08	KX675129	ITS2
Polyporus umbellatus	Chuqimen Medicinal Market,Chongqing,China	YC0018MT09	KX675130	ITS2
Poria cocos	Drugstore , Beijing , China	YC0135MT12	KX674753	ITS2
Poria cocos	Drugstore , Beijing , China	YC0135MT13	KX674754	ITS2
Poria cocos	Drugstore , Beijing , China	YC0135MT14	KX675005	ITS2
Prunella vulgaris var. lilacina	Botanical garden, University of Toyama, Japan	JP031MT01	KU174614	ITS2
Prunella vulgaris var. lilacina	Botanical garden, University of Toyama, Japan	JP031MT02	KU174615	ITS2
Prunella vulgaris var. lilacina	Botanical garden, University of Toyama, Japan	JP031MT03	KU174616	ITS2

Prunus armeniaca	The institute of medicinal plant development,Beijing,China	PS1123MT02	KX674755	ITS2
Prunus armeniaca	Kuancheng, Hebei，China	YC0428MT03	KX674756	ITS2
Prunus armeniaca	Kuancheng, Hebei，China	YC0428MT04	KX674920	ITS2
Prunus armeniaca var. ansu	Drug store, Tianjin，China	YC0582MT01	KX675006	ITS2
Prunus armeniaca var. ansu	National Institute for Food and Drug Control，Beijing，China	YC0582MT04	KX675007	ITS2
Prunus armeniaca var. ansu	Kuancheng, Hebei，China	YC0582MT02	KX675008	ITS2
Prunus davidiana	The institute of medicinal plant development,Beijing,China	PS1109MT03	KX674810	ITS2
Prunus davidiana	Nanchuan, Chongqing, China	YC0080MT08	KX674811	ITS2
Prunus davidiana	Nanchuan, Chongqing, China	YC0080MT09	KX675054	ITS2
Prunus persica	Putian, Fujian，China	PS1117MT02	KX674812	ITS2
Prunus persica	The institute of medicinal plant development,Beijing,China	YC0055MT24	KX674813	ITS2
Prunus persica	The institute of medicinal plant development,Beijing,China	YC0055MT26	KX675055	ITS2
Prunus sibirica	The institute of medicinal plant development,Beijing,China	PS1121MT03	KX674919	ITS2

Prunus sibirica	Kuancheng, Hebei，China	YC0583MT01	KX675009	ITS2
Prunus sibirica	Drugstore , Beijing , China	YC0583MT02	KX675010	ITS2
Pueraria lobata	Yunlin, Guangxi,China	YC0162MT08	KX674778	ITS2
Pueraria lobata	Yunlin, Guangxi,China	YC0162MT09	KX674779	ITS2
Pueraria lobata	Yunlin, Guangxi,China	YC0162MT04	KX675033	ITS2
Quercus acutissima	Nanchuan, Chongqing, China	JP033MT01	KU174547	ITS2
Quercus acutissima	Nanchuan, Chongqing, China	JP033MT02	KU174548	ITS2
Quercus mongolica var. crispula	Changbaishan, Jilin, China	JP034MT01	KU174549	ITS2
Quercus mongolica var. crispula	Changbaishan, Jilin, China	JP034MT02	KU174550	ITS2
Quercus mongolica var. crispula	Changbaishan, Jilin, China	JP034MT03	KU174554	ITS2
Quercus serrata	\	\	JF980309	ITS2
Quercus serrata	Beijing Batanical Garden, Beijing, China	JP032MT01	KU174551	ITS2
Quercus serrata	Beijing Batanical Garden, Beijing, China	JP032MT02	KU174552	ITS2
Quercus variabilis	Beijing Batanical Garden, Beijing, China	JP035MT01	KU174544	ITS2
Quercus variabilis	Beijing Batanical Garden, Beijing, China	JP035MT02	KU174545	ITS2
Quercus variabilis	Nanchuan, Chongqing, China	JP035MT03	KU174546	ITS2
Rehmannia glutinosa	Drugstore , Beijing , China	YC0129MT25	KX674757	ITS2
Rehmannia glutinosa	Drugstore , Beijing , China	YC0129MT30	KX674758	ITS2
Rehmannia glutinosa	Drugstore , Beijing , China	YC0129MT29	KX675011	ITS2

Rheum officinale	Bijie, Guizhou，China	YC0349MT37	KX674972	ITS2
Rheum officinale	Shennongjia, Hubei，China	YC0349MT06	KX674977	ITS2
Rheum officinale	Shennongjia, Hubei，China	YC0349MT02	KX675106	ITS2
Rheum palmatum	Nuoergai, Sichuan，China	YC0782MT26	KX674975	ITS2
Rheum palmatum	Maerkang, Sichuan，China	YC0782MT29	KX674976	ITS2
Rheum palmatum	Maerkang, Sichuan，China	YC0782MT28	KX675107	ITS2
Rheum tanguticum	Bijie, Guizhou，China	YC0783MT21	KX674973	ITS2
Rheum tanguticum	Banma, Qinghai,China	YC0783MT09	KX674974	ITS2
Rheum tanguticum	Banma, Qinghai,China	YC0783MT08	KX675108	ITS2
Rosa multiflora	Nanchuan, Chongqing, China	JP036MT04	KU174553	ITS2
Rosa multiflora	Fukuoka University, Fukuoka, Japan	JP036MT02	KU174555	ITS2
Rosa multiflora	Fukuoka University, Fukuoka, Japan	JP036MT03	KU174556	ITS2
Saposhnikovia divaricata	Bozhou Medicinal Market,Anhui,China	YC0003MT08	KX674841	ITS2
Saposhnikovia divaricata	Yunlin, Guangxi,China	YC0003MT01	KX674842	ITS2
Saposhnikovia divaricata	Yunlin, Guangxi,China	YC0003MT02	KX675121	ITS2
Schisandra chinensis	Bozhou Medicinal Market,Anhui,China	YC0092MT45	KX674855	ITS2
Schisandra chinensis	Changchun, Jilin, China	YC0092MT30	KX674856	ITS2
Schisandra chinensis	Xinhehuachi Medicinal Market,Sichuan,China	YC0092MT01	KX674907	ITS2
Schizonepeta tenuifolia	Changchun, Jilin, China	JL-28-1	KX674966	ITS2

Schizonepeta tenuifolia	Anguo Medicinal Market,Hebei,China	YC0176MT21	KX674967	ITS2
Schizonepeta tenuifolia	Bozhou Medicinal Market,Anhui,China	YC0176MT22	KX674968	ITS2
Scopolia carniolica	\	\	AY478410	ITS2
Scopolia carniolica	\	\	AY478411	ITS2
Scopolia japonica	\	\	AY478412	ITS2
Scopolia japonica	\	\	AY478413	ITS2
Scopolia japonica	University of Toyama, Toyama, Japan	JP037MT01	KX714226	ITS2
Scopolia parviflora	\	\	AY478404	ITS2
Scopolia parviflora	\	\	AY478405	ITS2
Scopolia parviflora	\	\	AY478406	ITS2
Scutellaria baicalensis	Aershan, Neimenggu, China	YC0039MT17	KX674993	ITS2
Scutellaria baicalensis	Changchun, Jilin, China	YC0039MT21	KX674994	ITS2
Scutellaria baicalensis	Changchun, Jilin, China	YC0039MT20	KX674995	ITS2
Sesamum indicum	Yunlin, Shaanxi， China	YC0198MT09	KX674863	ITS2
Sesamum indicum	Xinhehuachi Medicinal Market,Sichuan,China	YC0198MT03	KX674992	ITS2
Sesamum indicum	Anguo Medicinal Market,Hebei,China	YC0198MT01	KX675137	ITS2
Sinomenium acutum	Nanyang, Henan , China	YC0182MT14	KX674871	ITS2
Sinomenium acutum	Nanyang, Henan , China	YC0182MT12	KX674872	ITS2
Sinomenium acutum	Nanyang, Henan , China	YC0182MT14	KX674969	ITS2

Sophora flavescens	Shennongjia, Hubei , China	YC0212MT19	KX674878	ITS2
Sophora flavescens	Anguo Medicinal Market,Hebei,China	YC0212MT08	KX674879	ITS2
Sophora flavescens	Anguo Medicinal Market,Hebei,China	YC0212MT09	KX674933	ITS2
Strychnos nux-vomica	Xishuangbanna, Yunnan , China	PS0891MT02	KX674945	ITS2
Strychnos nux-vomica	National Institute for Food and Drug Control，Beijing，China	FDC149	KX674946	ITS2
Strychnos nux-vomica	Xishuangbanna, Yunnan , China	YC0616MT01	KX675063	ITS2
Swertia japonica	The University of Tokyo, Tokyo, Japan	JP042MT01	KU174602	ITS2
Swertia japonica	Tsumura & Co., Japan	JP042MT04	KU174603	ITS2
Swertia japonica	The University of Tokyo, Tokyo, Japan	JP042MT02	KU174604	ITS2
Tribulus terrestris	Bozhou Medicinal Market,Anhui,China	PS0490MT04	KX674911	ITS2
Tribulus terrestris	Nanyang, Henan，China	YC0033MT08	KX675034	ITS2
Tribulus terrestris	Shihezi, Xinjiang, China	YC0033MT09	KX675035	ITS2
Trichosanthes kirilowii	The institute of medicinal plant development,Beijing,China	PS0463MT01	KX674844	ITS2
Trichosanthes kirilowii	Anguo Medicinal Market,Hebei,China	YC0042MT11	KX674845	ITS2
Trichosanthes kirilowii	Bozhou Medicinal Market,Anhui,China	YC0042MT28	KX675138	ITS2
Trichosanthes kirilowii var. japonica	University of Toyama, Toyama, Japan	JP044MT01	KU174646	ITS2
Trichosanthes kirilowii var. japonica	University of Toyama, Toyama, Japan	JP044MT02	KU174647	ITS2

Trichosanthes kirilowii var. japonica	University of Toyama, Toyama, Japan	JP044MT03	KU174648	ITS2
Uncaria macrophylla	Xishuangbanna, Yunnan , China	PS1038MT04	KX674759	ITS2
Uncaria macrophylla	Nanjing, Jiangsu , China	YC0577MT01	KX675012	ITS2
Uncaria macrophylla	Nanjing, Jiangsu , China	YC0577MT02	KX675013	ITS2
Uncaria rhynchophylla	Guangzhou, Guangdong，China	YC0578MT05	KX674760	ITS2
Uncaria rhynchophylla	Nanchuan, Chongqing, China	YC0578MT04	KX674761	ITS2
Uncaria rhynchophylla	Nanchuan, Chongqing, China	YC0578MT02	KX675014	ITS2
Uncaria sinensis	Nanchuan, Chongqing, China	PS1039MT01	KX674762	ITS2
Uncaria sinensis	Nanchuan, Chongqing, China	YC0579MT03	KX674763	ITS2
Uncaria sinensis	Nanchuan, Chongqing, China	YC0579MT04	KX675015	ITS2
Valeriana fauriei	University of Toyama, Toyama, Japan	JP021MT01	KU174632	ITS2
Valeriana fauriei	Toyama, Japan	JP021MT02	KU174633	ITS2
Zanthoxylum piperitum	Toyama, Japan	JP043MT02	KU174565	ITS2
Zanthoxylum piperitum	Toyama, Japan	JP043MT03	KU174566	ITS2
Zanthoxylum piperitum	The University of Tokyo, Tokyo, Japan	JP043MT05	KU174567	ITS2
Zingiber officinale	Nanning, Guangxi，China	PS0523MT01	KX674913	ITS2
Zingiber officinale	Nanning, Guangxi，China	PS0523MT03	KX674914	ITS2
Zingiber officinale	Nanning, Guangxi，China	PS0523MT03	KX675096	ITS2
Ziziphus jujuba var. spinosa	Yunlin, Shaanxi，China	PS1339MT02	KX674814	ITS2

Ziziphus jujuba var. spinosa	Bozhou Medicinal Market,Anhui,China	YC0045MT04	KX674815	ITS2
Ziziphus jujuba var. spinosa	Chuqimen Medicinal Market,Chongqing,China	YC0045MT05	KX674923	ITS2
Areca catechu	Anguo Medicinal Market,Hebei,China	YC0070MT02	KX675147	*psbA-trnH*
Areca catechu	Xinhehuachi Medicinal Market,Sichuan,China	YC0070MT01	KX675163	*psbA-trnH*
Areca catechu	Yulin, Guangxi, China	YC0070MT02	KX675164	*psbA-trnH*
Cinnamomum cassia	Fuzhou, Fujian, China	YC0077MT19	KX675171	*psbA-trnH*
Cinnamomum cassia	Bozhou Medicinal Market,Anhui,China	YC0077MT01	KX675172	*psbA-trnH*
Cinnamomum cassia	Bozhou Medicinal Market,Anhui,China	YC0077MT01	KX675173	*psbA-trnH*
Dioscorea batatas	Nanyang, Henan,China	YC0004MT24	KX675139	*psbA-trnH*
Dioscorea batatas	Nanyang, Henan,China	YC0004MT25	KX675140	*psbA-trnH*
Dioscorea batatas	Nanyang, Henan,China	YC0004MT26	KX675141	*psbA-trnH*
Dioscorea japonica	Lushan Mountain, Jiangxi,China	YC0604MT07	KX675153	*psbA-trnH*
Dioscorea japonica	Liuyang, Hunan,China	YC0604MT01	KX675154	*psbA-trnH*
Dioscorea japonica	Liuyang, Hunan,China	YC0604MT02	KX675155	*psbA-trnH*
Lindera aggregata	Bozhou, Anhui, China	PS1630MT01	KX675142	*psbA-trnH*
Lindera aggregata	Drugstore, Beijing, China	YC0284MT03	KX675143	*psbA-trnH*
Lindera aggregata	Drugstore, Beijing, China	YC0284MT04	KX675161	*psbA-trnH*
Magnolia biondii	Guangdong, China	YC0117MT21	KX675148	*psbA-trnH*

Magnolia biondii	Bozhou Medicinal Market,Anhui,China	YC0117MT08	KX675149	*psbA-trnH*
Magnolia biondii	Bozhou Medicinal Market,Anhui,China	YC0117MT10	KX675165	*psbA-trnH*
Magnolia denudata	South China Botanical Garden，Guangdong，China	YC0621MT03	KX675166	*psbA-trnH*
Magnolia denudata	South China Botanical Garden，Guangdong，China	YC0621MT02	KX675167	*psbA-trnH*
Magnolia denudata	South China Botanical Garden，Guangdong，China	YC0621MT01	KX675168	*psbA-trnH*
Magnolia kobus	University of Toyama, Toyama, Japan	JP026MT01	KU174537	*psbA-trnH*
Magnolia kobus	University of Toyama, Toyama, Japan	JP026MT02	KU174538	*psbA-trnH*
Magnolia kobus	Botanical garden, Fukuoka University, Japan	JP026MT03	KU174540	*psbA-trnH*
Magnolia obovata	\	\	AB021029	*psbA-trnH*
Magnolia obovata	\	\	AB728567	*psbA-trnH*
Magnolia officinalis var. biloba/Magnolia officinalis	National Institute for Food and Drug Control，Beijing，China	YC0029MT03	KX675145	*psbA-trnH*
Magnolia officinalis var. biloba/Magnolia officinalis	Bozhou Medicinal Market,Anhui,China	YC0029MT10	KX675146	*psbA-trnH*
Magnolia officinalis var. biloba/Magnolia officinalis	Bozhou Medicinal Market,Anhui,China	YC0029MT14	KX675156	*psbA-trnH*
Magnolia salicifolia	University of Toyama, Toyama, Japan	JP025MT01	KU174539	*psbA-trnH*
Magnolia salicifolia	University of Toyama, Toyama, Japan	JP025MT02	KU174535	*psbA-trnH*
Magnolia salicifolia	University of Toyama, Toyama, Japan	JP025MT03	KU174536	*psbA-trnH*
Magnolia sprengeri	Beijing Batanical Garden, Beijing, China	PS3154MT01	KX675150	*psbA-trnH*

Magnolia sprengeri	Bozhou Medicinal Market,Anhui,China	YC0622MT01	KX675169	*psbA-trnH*
Magnolia sprengeri	Bozhou Medicinal Market,Anhui,China	YC0622MT01	KX675170	*psbA-trnH*
Myristica fragrans	Xinglong Tropical Medicinal Botanical Garden，Hainan,China	YC0184MT10	KX675158	*psbA-trnH*
Myristica fragrans	Xishuangbanna, Yunnan，China	YC0184MT16	KX675159	*psbA-trnH*
Myristica fragrans	Drugstore, Beijing, China	YC0184MT17	KX675160	*psbA-trnH*
Polygonatum cyrtonema	Sanming, Fujian, China	YC0036MT02	KX675151	*psbA-trnH*
Polygonatum cyrtonema	Sanming, Fujian, China	YC0036MT06	KX675174	*psbA-trnH*
Polygonatum cyrtonema	Shennongjia, Hubei,China	YC0036MT04	KX675175	*psbA-trnH*
Polygonatum kingianum	Nanchuan, Chongqing, China	YC0073MT05	KX675176	*psbA-trnH*
Polygonatum kingianum	Bijie, Guizhou, China	YC0073MT04	KX675177	*psbA-trnH*
Polygonatum kingianum	Bijie, Guizhou, China	YC0073MT03	KX675178	*psbA-trnH*
Polygonatum sibiricum	Nanchuan, Chongqing, China	YC0088MT09	KX675152	*psbA-trnH*
Polygonatum sibiricum	Anguo Medicinal Market,Hebei,China	YC0088MT01	KX675179	*psbA-trnH*
Polygonatum sibiricum	Anguo Medicinal Market,Hebei,China	YC0088MT01	KX675180	*psbA-trnH*
Smilax glabra	South China Botanical Garden，Guangdong，China	YC0249MT15	KX675144	*psbA-trnH*
Smilax glabra	Ganzhou, Jiangxi,China	YC0249MT05	KX675157	*psbA-trnH*
Smilax glabra	Ganzhou, Jiangxi,China	YC0249MT04	KX675162	*psbA-trnH*

Table S2 Identification results of the 100 test samples

Label Species Name	Sample ID	Best Match	Synonyms
Aconitum carmichaelii	ZL-145-01	*Aconitum carmichaelii*	
Astragalus membranaceus	JPHF099	*Astragalus membranaceus*	*Astragalus mongholicus*
Atractylodes japonica	ZL-077-01	*Atractylodes japonica*	*Atractylodes lancea*
Atractylodes japonica	ZL-077-02	*Atractylodes japonica*	*Atractylodes lancea*
Atractylodes japonica	ZL-077-03	*Atractylodes japonica*	*Atractylodes lancea*
Atractylodes lancea	JPBA007A	*Atractylodes lancea*	*Atractylodes japonica*
Atractylodes lancea	JPHF048	*Atractylodes lancea*	*Atractylodes japonica*
Akebia trifoliata	JPHF098	*Akebia trifoliata*	
Alisma orientale	JPHF106	*Alisma orientale*	
Angelica dahurica	JPHF096	*Angelica dahurica*	
Aralia cordata	JPHF100	*Aralia cordata*	
Arctium lappa	JPHF054	*Arctium lappa*	
Artemisia capillaris	JPHF076	*Artemisia capillaris*	
Astragalus membranaceus var. mongholicus	ZL-023-01	*Astragalus membranaceus var. mongholicus*	*Astragalus membranaceus*
Astragalus membranaceus var. mongholicus	ZL-023-02	*Astragalus membranaceus var. mongholicus*	
Asarum sieboldii	JPHF093	*Asarum sieboldii*	
Benincasa hispida	JPHF039	*Benincasa hispida*	

Bupleurum falcatum	JPHF105	*Bupleurum falcatum*	
Bupleurum falcatum	ZL-020-01	*Bupleurum falcatum*	
Cannabis sativa	JPHF025	*Cannabis sativa*	
Carthamus tinctorius	JPHF028	*Carthamus tinctorius*	
Cassia tora (C. obtusifolia)	JPHF034	*Cassia obtusifolia*	
Citrus reticulata	JPHF082	*Citrus reticulata*	*Citrus unshiu*
Cimicifuga dahurica	JPHF042	*Cimicifuga dahurica*	
Cnidium monnieri	JPHF038	*Cnidium monnieri*	
Coix lacryma-jobi var. mayuen	JPHF047	*Coix lacryma-jobi var. mayuen*	
Corydalis yanhusuo	JPHF045	*Corydalis yanhusuo*	
Dolichos lablab	JPHF053	*Dolichos lablab*	
Eriobotrya japonica	JPHF033	*Eriobotrya japonica*	
Foeniculum vulgare	JPHF056	*Foeniculum vulgare*	
Geranium thunbergii	JPBA037A	*Geranium thunbergii*	
Geranium thunbergii	JPBA037B	*Geranium thunbergii*	
Glehnia littoralis	ZL-022-01	*Glehnia littoralis*	
Glycyrrhiza uralensis	JPHF024	*Glycyrrhiza uralensis*	
Glycyrrhiza uralensis	ZL-013-01	*Glycyrrhiza uralensis*	
Glycyrrhiza uralensis	ZL-013-02	*Glycyrrhiza uralensis*	

Glycyrrhiza uralensis	ZL-013-03	*Glycyrrhiza uralensis*
Leonurus japonicus	JPHF107	*Leonurus japonicus*
Lithospermum erythrorhizon	JPHF111	*Lithospermum erythrorhizon*
Mentha arvensis var.piperascens	JPHF109	*Mentha arvensis var. piperascens*
Morus alba	JPHF016	*Morus alba*
Nelumbo nucifera	JPHF023	*Nelumbo nucifera*
Ophiopogon japonicus	ZL-075-01	*Ophiopogon japonicus*
Ophiopogon japonicus	ZL-075-02	*Ophiopogon japonicus*
Paeonia lactiflora	JPHF018	*Paeonia lactiflora*
Paeonia lactiflora	JPHF062	*Paeonia lactiflora*
Paeonia lactiflora	ZL-001-01	*Paeonia lactiflora*
Paeonia lactiflora	ZL-001-02	*Paeonia lactiflora*
Paeonia suffruticosa	JPHF020	*Paeonia suffruticosa*
Paeonia suffruticosa	ZL-006-01	*Paeonia suffruticosa*
Panax ginseng	JPHF021	*Panax ginseng*
Panax ginseng	ZL-015-01	*Panax ginseng*
Panax ginseng	ZL-015-02	*Panax ginseng*
Panax ginseng	ZL-015-03	*Panax ginseng*
Panax japonicus	JPHF064	*Panax japonicus*

Peucedanum praeruptorum	JPHF071	*Peucedanum praeruptorum*
Phellodendron amurense	JPHF086	*Phellodendron amurense*
Plantago asiatica	JPHF030	*Plantago asiatica*
Platycodon grandiflorum	ZL-025-01	*Platycodon grandiflorum*
Platycodon grandiflorum	ZL-025-02	*Platycodon grandiflorum*
Platycodon grandiflorum	ZL-025-03	*Platycodon grandiflorum*
Pogostemon cablin	JPHF027	*Pogostemon cablin*
Polygala tenuifolia	JPHF008	*Polygala tenuifolia*
Pueraria lobata	JPHF002	*Pueraria lobata*
Saposhnikovia divaricata	ZL-031-01	*Saposhnikovia divaricata*
Saposhnikovia divaricata	ZL-031-02	*Saposhnikovia divaricata*
Saposhnikovia divaricata	ZL-031-03	*Saposhnikovia divaricata*
Schisandra chinensis	JPHF017	*Schisandra chinensis*
Schisandra chinensis	ZL-043-02	*Schisandra chinensis*
Schisandra chinensis	ZL-043-03	*Schisandra chinensis*
Schizonepeta tenuifolia	JPHF029	*Schizonepeta tenuifolia*
Scutellaria baicalensis	JPHF013	*Scutellaria baicalensis*
Sophora flavescens	JPHF061	*Sophora flavescens*
Euodia rutaecarpa	JPHF068	*Euodia rutaecarpa*

Tribulus terrestris	JPHF059	*Tribulus terrestris*
Uncaria rhynchophylla	JPHF074	*Uncaria rhynchophylla*
Ziziphus jujuba var. spinosa	JPBA018	*Ziziphus jujuba var. spinosa*
Ziziphus jujuba var. spinosa	JPHF067	*Ziziphus jujuba var. spinosa*
Angelica acutiloba var. sugiyamae	JPFS002B	*Angelica acutiloba var. sugiyamae,Angelica acutiloba*
Angelica acutiloba	ZL-002-01	*Angelica acutiloba,Angelica acutiloba var. sugiyamae*
Angelica acutiloba	ZL-002-02	*Angelica acutiloba,Angelica acutiloba var. sugiyamae*
Angelica acutiloba	ZL-002-03	*Angelica acutiloba,Angelica acutiloba var. sugiyamae*
Chrysanthemum indicum	JPHF081	*Chrysanthemum indicum,Chrysanthemum morifolium*
Clematis hexapetala	JPHF041	*Clematis hexapetala,Clematis manshurica*
Clematis manshuria	ZL-083-01	*Clematis manshurica,Clematis hexapetala,Clematis chinensis*
Clematis manshuria	ZL-083-02	*Clematis manshurica,Clematis hexapetala,Clematis chinensis*
Ephedra sinica	JPHF103	*Ephedra sinica,Ephedra intermedia*
Ephedra sinica	ZL-037-01	*Ephedra sinica,Ephedra intermedia*
Ephedra sinica	ZL-037-02	*Ephedra sinica,Ephedra intermedia*
Ephedra sinica	ZL-037-03	*Ephedra sinica,Ephedra intermedia*
Epimedium grandiflorum var. thunbergianum	JPBA049A	*Epimedium grandiflorum var. thunbergianum,Epimedium koreanum*
Epimedium grandiflorum var. thunbergianum	JPBA049B	*Epimedium grandiflorum var. thunbergianum,Epimedium koreanum*
Gentiana scabra	ZL-080-01	*Gentiana scabra,Gentiana manshurica,Gentiana triflora*

Gentiana scabra	ZL-080-02	*Gentiana scabra,Gentiana manshurica,Gentiana triflora*
Perilla frutescens var. crispa	JPHF110	*Perilla frutescens var. crispa,Perilla frutescens var. acuta*
Perilla frutescens	ZL-091-01	*Perilla frutescens var. acuta,Perilla frutescens var. crispa*
Perilla frutescens	ZL-091-02	*Perilla frutescens var. acuta,Perilla frutescens var. crispa*
Perilla frutescens	ZL-091-03	*Perilla frutescens var. acuta,Perilla frutescens var. crispa*
Prunus armeniaca	JPHF113	*Prunus armeniaca,Prunus sibirica,Prunus armeniaca var. ansu*
Prunus armeniaca var. ansu	ZL-046-01	*Prunus armeniaca var. ansu,Prunus sibirica,Prunus armeniaca*

Table S3 Stable variable sites in ITS2 sequences between *A. acutiloba* and *A. sinensis*

Position (bp)	7	12	21	30	34	36	38	41	45	47	51	57	64	76	83	96	101	102
A. sinensis	A	T	-	T	C	T	G	C	A	T	A	G	T	G	T	C	G	T
A. acutiloba	G	-	A	A	-	A	A	T	G	C	T	-	C	A	C	G	A	C

Position (bp)	125	131	145	152	163	172	184	191	205	215	221	228
A. sinensis	T	T	T	A	C	G	T	A	G	T	A	A
A. acutiloba	C	C	G	T	T	T	C	C	A	C	G	C

Table S4 Identification efficacy of the test samples from the two studies.

	Total involved species	Identified at species level	Identification efficacy %
JP	66	54	81.8
AP	90	69	76.7

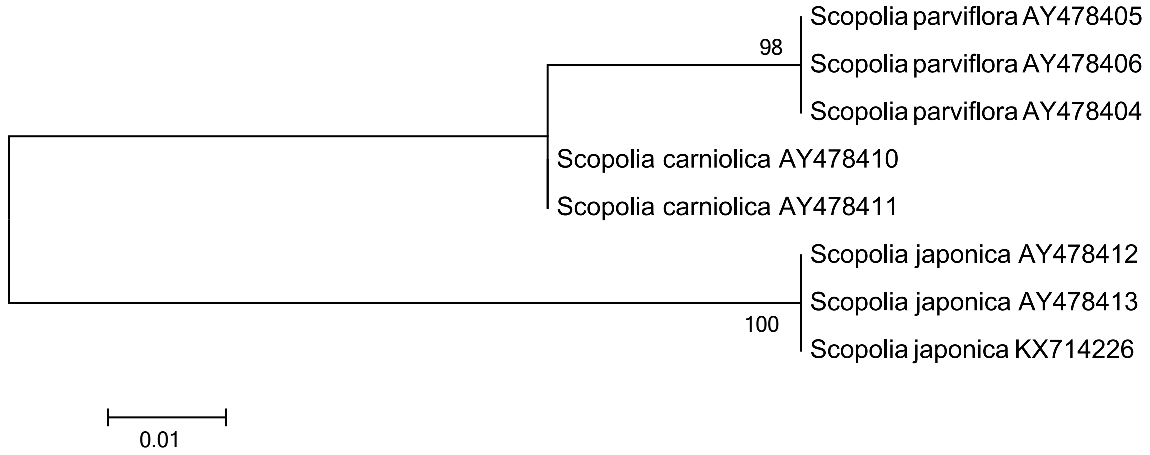

Fig. S1 NJ tree of *Scopolia* based on ITS2 sequences
